# Supplementary material for: Genetic associations with ratios between protein levels detect new pQTLs and reveal protein-protein interactions
Source: Cell Genom. 2024 Feb 26;4(3):100506. doi: 10.1016/j.xgen.2024.100506 (PMC10943581; doi:10.1016/j.xgen.2024.100506)
Supplement: Document S1. Figures S1—S5 [file mmc1.pdf]

Cell Genomics, Volume *4*

Supplemental information

Genetic associations with ratios between protein levels detect new pQTLs and reveal protein-protein interactions

Karsten Suhre

SUPPLEMENTAL MATERIAL

Genetic associations with ratios between protein levels detect new pQTLs and reveal protein-protein interactions.

Karsten Suhre[1,2,*]

[1] Bioinformatics Core, Weill Cornell Medicine-Qatar, Education City, 24144 Doha, Qatar

[2] Department of Biophysics and Physiology, Weill Cornell Medicine, New York, NY, U.S.A.

[*] Correspondence to K.S. (kas2049@qatar-med.cornell.edu)

SUPPLEMENTAL FIGURES

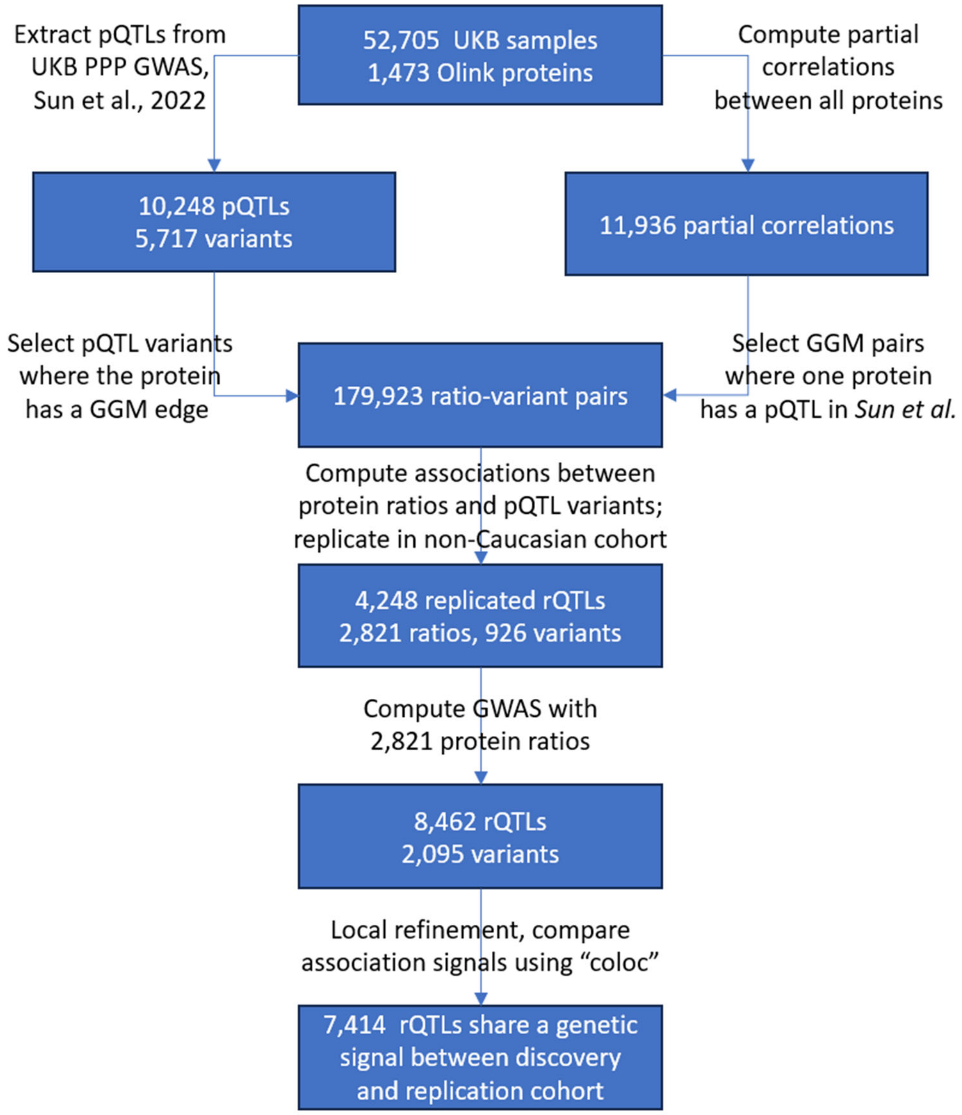

Figure S1: Flowchart of the study, related to Figures 1-2

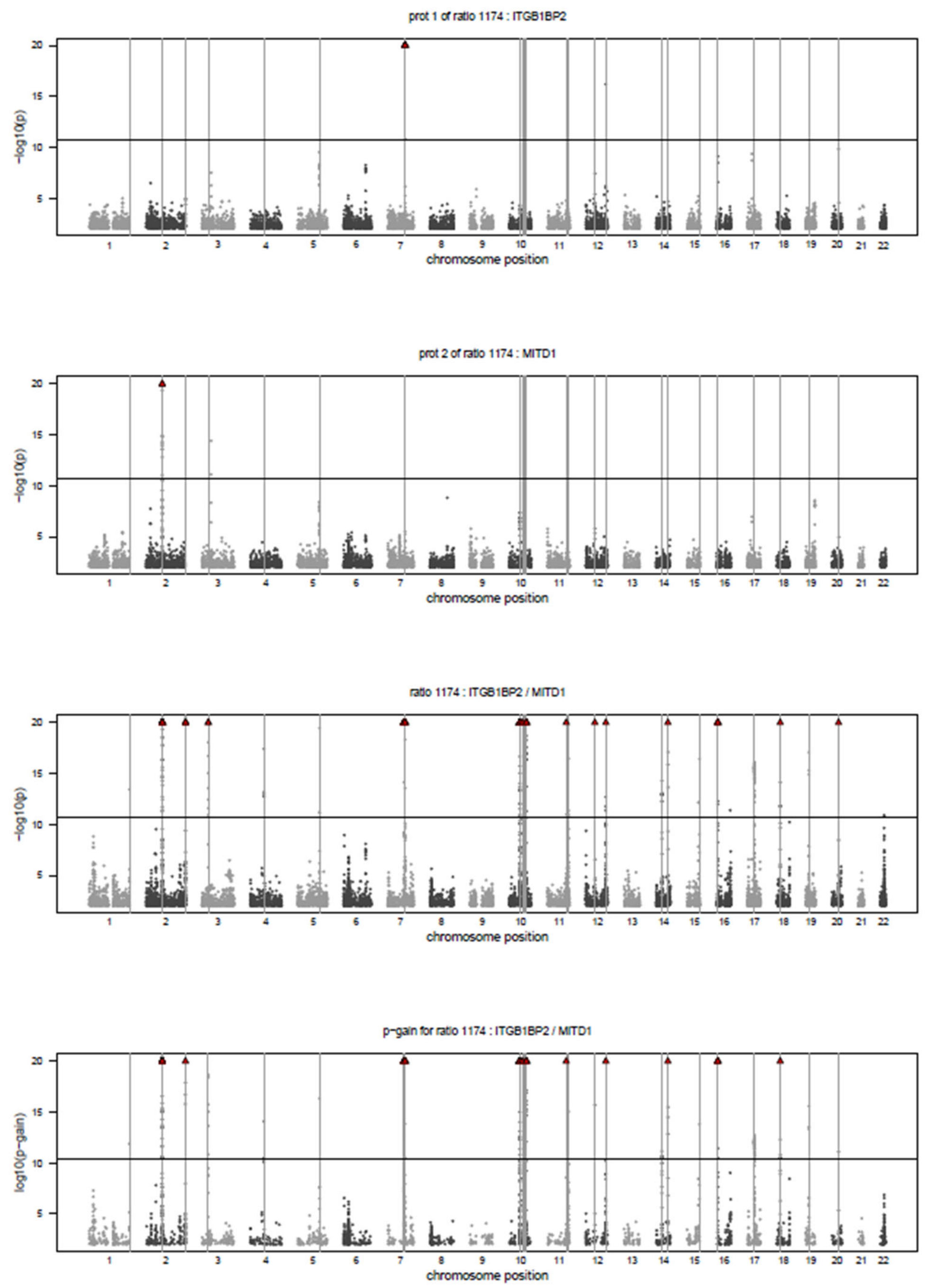

Figure S2: Example of Manhattan plots for a ratio, related to Figures 1-2. This Figure explains how by using ratios novel genetic loci can be uncovered. Plotted are the associations of the two individual proteins (ITGB1BP2 and MTD1), the ratio (ITGB1BP2 / MTD1), and the p-gain of the ratio using array genotype data; Similar Manhattan plots are available in PDF format for the 2,821 ratios on FigShare (doi:10.6084/m9.figshare.23695398).

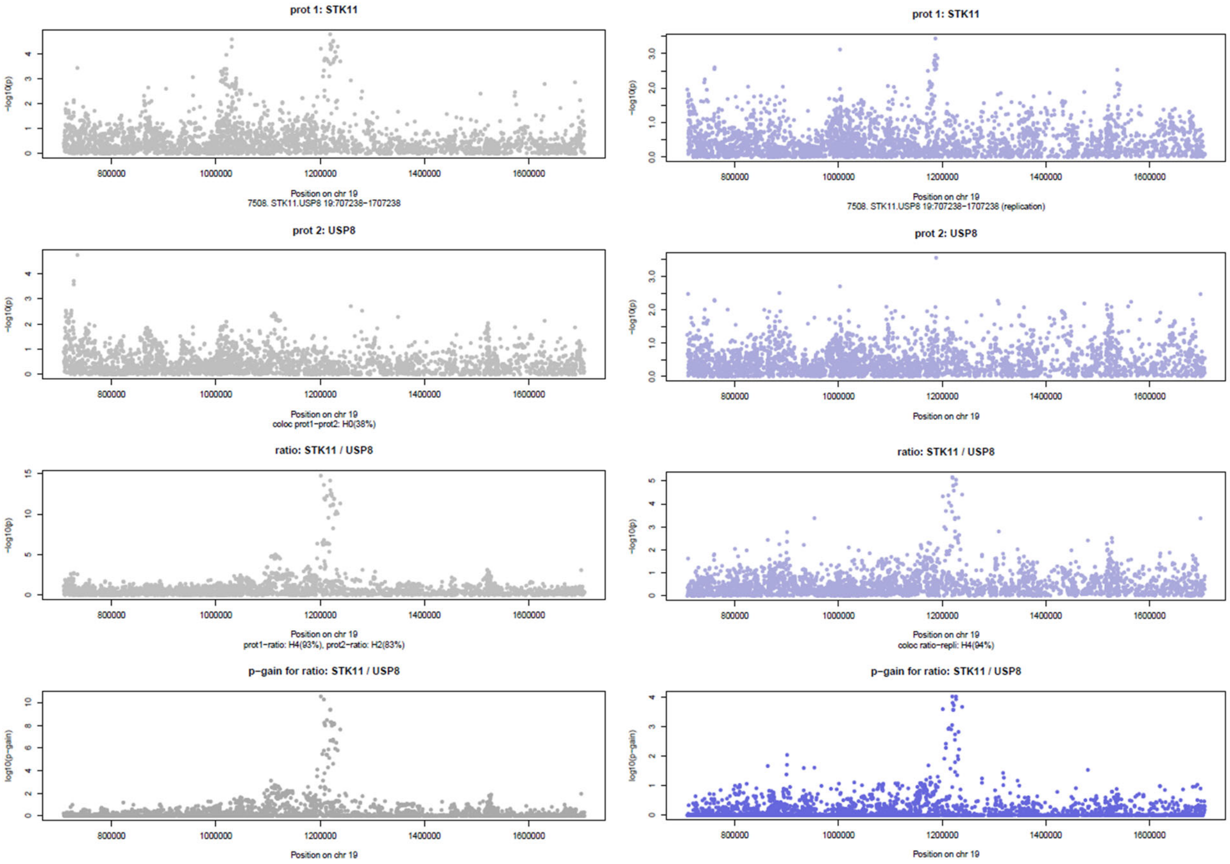

(a) (b)

Figure S3: Example of regional association plots for rQTLs, related to Figures 1-2. This Figure explains how by using ratios a genetic signal can emerge from the noise. Plotted are the associations of the two individual proteins (STK11 and USP8), the ratio (STK11 / USP8), and the p-gain of the ratio using imputed genotype data +/-500kb around the variant rs3764640 for the discovery (a) and the replication cohort (b); The subtitles indicate the most likely *coloc* hypotheses regarding the similarity between the relevant genetic signals; Similar regional association plots together with the full summary statistics used in these plots for 8,462 rQTLs are available in PDF format on FigShare (doi:10.6084/m9.figshare.23695398).

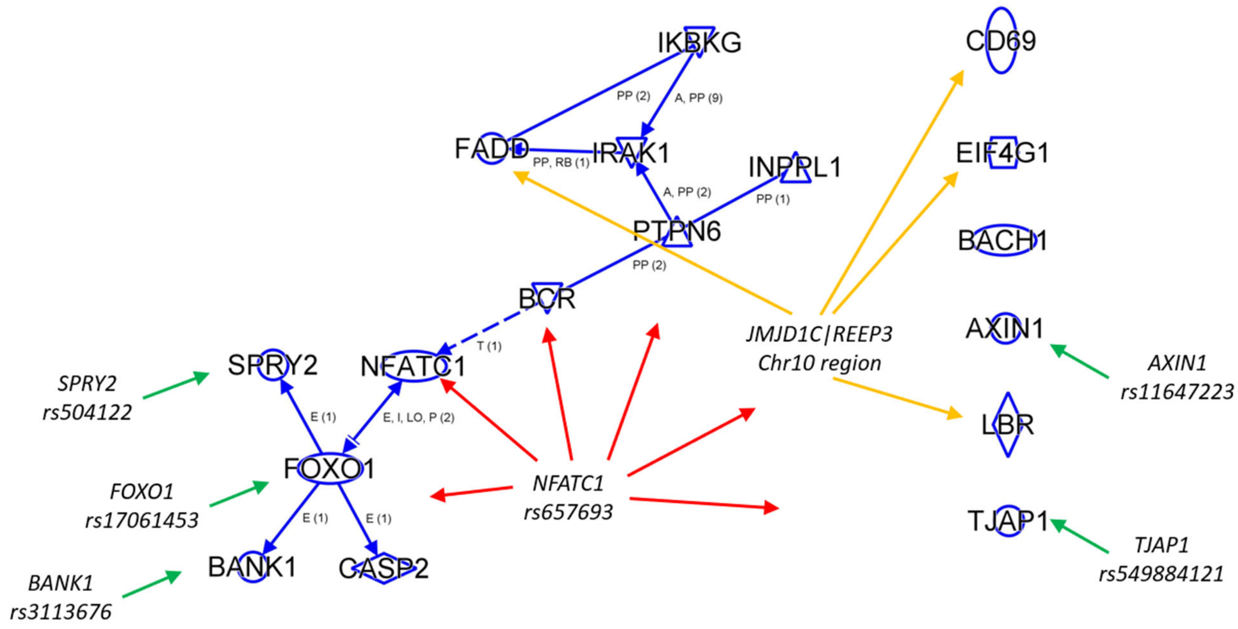

Figure S4: NFATC1 network, related to Figures 1-2. Protein-protein interactions obtained using Ingenuity Pathway Analysis (IPA)'s connect function with default settings (accessed 4 July 2023); Blue arrows indicate IPA interactions with the following abbreviations: A: activation, E: expression, I: inhibition, LO: localization, P: phosphorylation, PP: protein-protein interaction, RB: regulation of binding, T: transcription; Numbers in parentheses indicate multiple sources of evidence; Green arrows: rQTLs for the ratio of NFATC1 with the respective proteins at a *cis*-location; Orange: multiple variants on Chr10 around 65 MB associated with ratios between the listed proteins and NFATC1; Red: rs657693 at the *NFATC1* gene locus associated with all depicted proteins in a ratio with NFATC1 (details are in Table S4).

	CCL13	CCL8	CCL7	CCL2	CCL26	CXCL8	CCL11	CCL3	CCL14	CXCL6	TGFB1	PDGFA	HGF	THPO	TNFSF13	CXCL12	CXCL1	CCL17	CCL4	CXCL11	TNFSF14	IL7
CCL13	-230.0	542.9	500.1	0.0	0.0	0.0	0.0	0.0	0.0	0.0	75.7	66.0	0.0	57.8	28.8	20.5	0.0	0.0	0.0	4.2	10.7	0.0
CCL8	-542.9	179.8	0.0	-335.7	-291.1	-226.0	-106.0	-54.3	-98.1	-87.7	-54.1	0.0	-4.6	-36.0	-16.9	0.0	-3.1	-5.7	-14.5	0.0	0.0	0.0
CCL7	-500.1	0.0	337.3	-410.0	-274.5	-147.4	-122.7	-116.5	-108.2	0.0	-29.6	0.0	-57.9	0.0	-13.9	0.0	0.0	0.0	0.0	0.0	0.0	0.0
CCL2	0.0	335.7	410.0	-291.1	0.0	0.0	0.0	0.0	0.0	0.0	0.0	0.0	0.0	0.0	2.3	0.0	0.0	0.0	0.0	0.0	0.0	0.0
CCL26	0.0	291.1	274.5	0.0	-182.8	0.0	0.0	0.0	0.0	0.0	28.5	5.8	14.9	17.5	19.3	12.5	0.0	0.0	0.0	0.0	0.0	0.0
CXCL8	0.0	226.0	147.4	0.0	0.0	-89.4	0.0	0.0	0.0	0.0	8.0	0.0	0.0	1.5	8.4	0.0	0.0	0.0	0.0	0.0	0.0	0.0
CCL11	0.0	106.0	122.7	0.0	0.0	0.0	-33.8	0.0	0.0	0.0	0.0	0.0	0.0	0.0	0.0	0.0	0.0	0.0	0.0	0.0	0.0	0.0
CCL3	0.0	54.3	116.5	0.0	0.0	0.0	0.0	-14.5	0.0	0.0	0.0	0.0	0.0	0.0	1.4	0.0	0.0	0.0	0.0	0.0	0.0	0.0
CCL14	0.0	98.1	108.2	0.0	0.0	0.0	0.0	0.0	-31.5	0.0	0.0	0.0	0.0	0.0	5.2	0.0	0.0	0.0	0.0	0.0	0.0	0.0
CXCL6	0.0	87.7	0.0	0.0	0.0	0.0	0.0	0.0	0.0	-32.2	12.1	7.5	0.0	5.1	3.5	0.9	0.0	0.0	0.0	11.7	3.1	10.3
TGFB1	-75.7	54.1	29.6	0.0	-28.5	-8.0	0.0	0.0	0.0	-12.1	-0.2	0.0	0.0	0.0	0.1	0.0	-6.1	-5.4	-0.1	0.9	0.0	0.1
PDGFA	-66.0	0.0	0.0	0.0	-5.8	0.0	0.0	0.0	0.0	-7.5	0.0	-0.3	0.0	0.0	0.1	0.0	-3.3	-9.8	0.0	0.0	0.0	0.2
HGF	0.0	4.6	57.9	0.0	-14.9	0.0	0.0	0.0	0.0	0.0	0.0	0.0	-1.1	0.0	0.2	0.0	0.0	0.0	0.0	1.0	0.0	0.0
THPO	-57.8	36.0	0.0	0.0	-17.5	-1.5	0.0	0.0	0.0	-5.1	0.0	0.0	0.0	-0.4	0.1	0.0	-2.6	-5.4	0.0	0.7	0.0	0.0
TNFSF13	-28.8	16.9	13.9	-2.3	-19.3	-8.4	0.0	-1.4	-5.2	-3.5	-0.1	-0.1	-0.2	-0.1	0.2	-0.1	-2.1	-2.6	-0.9	0.0	-0.1	0.0
CXCL12	-20.5	0.0	0.0	0.0	-12.5	0.0	0.0	0.0	0.0	-0.9	0.0	0.0	0.0	0.0	0.1	-0.1	0.0	-1.1	0.0	0.1	0.0	0.0
CXCL1	0.0	3.1	0.0	0.0	0.0	0.0	0.0	0.0	0.0	0.0	6.1	3.3	0.0	2.6	2.1	0.0	-20.4	0.0	0.0	5.8	2.3	8.5
CCL17	0.0	5.7	0.0	0.0	0.0	0.0	0.0	0.0	0.0	0.0	5.4	9.8	0.0	5.4	2.6	1.1	0.0	-17.9	0.0	8.4	2.9	5.1
CCL4	0.0	14.5	0.0	0.0	0.0	0.0	0.0	0.0	0.0	0.0	0.1	0.0	0.0	0.0	0.9	0.0	0.0	0.0	-3.5	2.3	0.0	0.0
CXCL11	-4.2	0.0	0.0	0.0	0.0	0.0	0.0	0.0	0.0	-11.7	-0.9	0.0	-1.0	-0.7	0.0	-0.1	-5.8	-8.4	-2.3	2.0	-0.3	0.0
TNFSF14	-10.7	0.0	0.0	0.0	0.0	0.0	0.0	0.0	0.0	-3.1	0.0	0.0	0.0	0.0	0.1	0.0	-2.3	-2.9	0.0	0.3	-0.3	0.1
IL7	0.0	0.0	0.0	0.0	0.0	0.0	0.0	0.0	0.0	-10.3	-0.1	-0.2	0.0	0.0	0.0	0.0	-8.5	-5.1	0.0	0.0	-0.1	0.1

Figure S5: P-gain matrix for the association of rs12075 with all ratios between cytokines, related to Figures 1-2. SNP rs12075 (1:159175354:G:A) is an amino acid changing variant in ACKR1 aka DARC (c.125G>A, p.Gly42Asp) and defines the co-dominant Duffy blood type alleles Fy^a (Gly) and Fy^b (Asp). Limited to associations with -log10(p-value) > 10 or log10(p-gain) > 10 (full matrix in Supplementary Table 6); Values on the diagonal are -log10(p-value) for the single protein associations; Values in the off-diagonal cells are -log10(p-gain); The directionality of the associations with the copy number of the Fy^a allele are indicated by the sign and colored red (negative association) and green (positive association). Note that the -log10(p-value) for the ratios can be obtained by adding the log10(p-gain) of the ratio to the larger of the two -log10(p-value) of the single protein associations (full data in Table S9).
