# Supplementary material for: Genetic associations with ratios between protein levels detect new pQTLs and reveal protein-protein interactions
Source: Cell Genom. 2024 Feb 26;4(3):100506. doi: 10.1016/j.xgen.2024.100506 (PMC10943581; doi:10.1016/j.xgen.2024.100506)
Supplement: Document S3. Article plus supplemental information [file mmc12.pdf]

Cell Genomics

Article

Genetic associations with ratios between protein levels detect new pQTLs and reveal protein-protein interactions

Graphical abstract

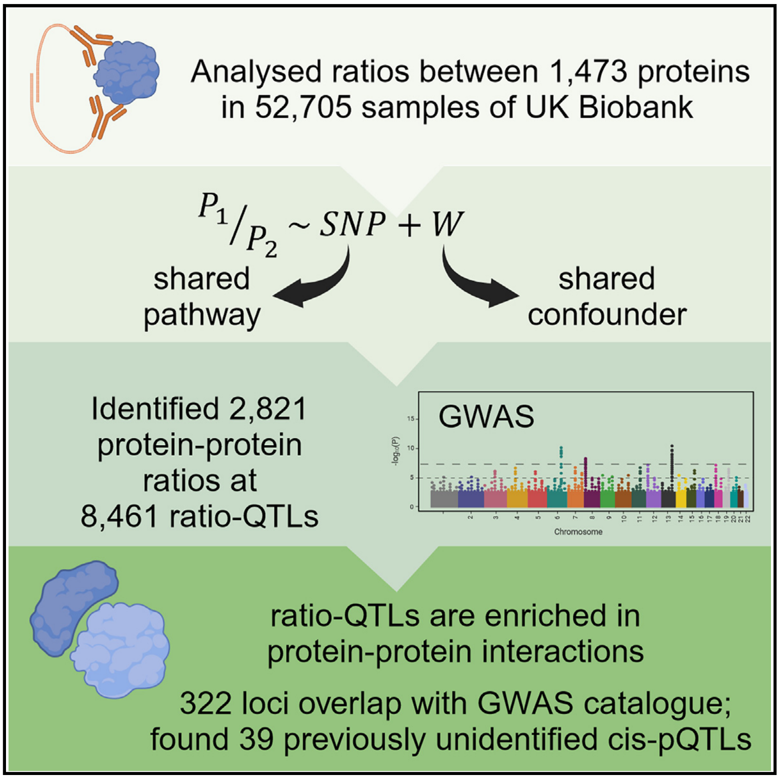

Highlights

- Use of ratios increased the number of proteogenomic loci by 25%
- Use of ratios reduced the number of Olink targets without a *cis*-pQTL by 13%
- Ratio QTLs are 7.6-fold enriched in established protein-protein interactions
- Theoretical development and generalization of the concept of ratios are warranted

Authors

Karsten Suhre

Correspondence

kas2049@qatar-med.cornell.edu

In brief

Protein quantitative trait loci (pQTLs) are a valuable resource for drug target development. Here, Suhre shows that a systematic analysis of ratios between protein pairs strengthens associations at known pQTL loci by several hundred orders of magnitude (p-gain), suggesting that these ratios reflect biological links between the implicated proteins.

Suhre, 2024, Cell Genomics *4*, 100506
March 13, 2024 © 2024 The Author(s).
https://doi.org/10.1016/j.xgen.2024.100506

CellPress

Cell Genomics

CellPress
OPEN ACCESS

Article

Genetic associations with ratios between protein levels detect new pQTLs and reveal protein-protein interactions

Karsten Suhre[1,2,3,*]
[1]Bioinformatics Core, Weill Cornell Medicine-Qatar, Education City, Doha 24144, Qatar
[2]Englander Institute for Precision Medicine, Weill Cornell Medicine, New York, NY 10021, USA
[3]Lead contact
*Correspondence: kas2049@qatar-med.cornell.edu
https://doi.org/10.1016/j.xgen.2024.100506

SUMMARY

Protein quantitative trait loci (pQTLs) are an invaluable source of information for drug target development because they provide genetic evidence to support protein function, suggest relationships between *cis*- and *trans*-associated proteins, and link proteins to disease endpoints. Using Olink proteomics data for 1,463 proteins measured in over 54,000 samples of the UK Biobank, we identified 4,248 associations with 2,821 ratios between protein levels (rQTLs). rQTLs were 7.6-fold enriched in known protein-protein interactions, suggesting that their ratios reflect biological links between the implicated proteins. Conducting a GWAS on ratios increased the number of discovered genetic signals by 24.7%. The approach can identify novel loci of clinical relevance, support causal gene identification, and reveal complex networks of interacting proteins. Taken together, our study adds significant value to the genetic insights that can be derived from the UKB proteomics data and motivates the wider use of ratios in large-scale GWAS.

INTRODUCTION

Large-scale studies of the blood circulating proteome leverage the natural variation in the general population to identify genetic and nongenetic factors that control blood protein levels.[1] Of particular interest for drug development are genome-wide association studies (GWASs) that identify protein quantitative trait loci (pQTLs), because they provide genetic evidence for a causal effect of the underlying variant, and hence the affected gene(s), on the levels of the associated protein(s) and their physiological effects. In cases of *cis*-pQTLs, in which the genetic variant is located in proximity of the gene coding for the associated pQTL protein, the effect is most likely through a causal variant that modifies transcription, translation, or stability of the *cis*-encoded protein. More complex, but also more rewarding in terms of potential biological insights, are *trans*-pQTLs because they suggest direct or indirect protein-protein interactions between the presumably causal *cis*-encoded protein and the associated *trans*-protein, which can extend into larger networks when multiple proteins are associated with the same variant and ideally also clinical endpoints of interest.

Such genetics-driven insights are of the highest value to pharmaceutical companies because they can inform drug target discovery and validation, generate hypotheses on modes of action, and suggest biomarkers for target engagement and efficacy. Early successes of pQTL studies[2–5] led to the creation of the UK Biobank Pharma Proteomics Project (UKB PPP) consortium, a precompetitive consortium of 13 biopharmaceutical companies that funded the measurement of over 54,000 UKB samples on the Olink Explore 1536 affinity proteomics platform. Olink uses a dual antibody binding technique, called the proximity extension assay, to quantify the abundance of almost 1,500 blood circulating proteins (Table S1). The UKB PPP consortium recently published initial results from a GWAS that identified over 10,000 pQTLs using this platform.[6] The Olink proteomics data were released in April 2023 to the public and can be accessed and analyzed using the DNAnexus UKB Research Analysis Platform (RAP) platform (ukbiobank.dnanexus.com). The aim in this paper was to explore new methods to enhance pQTL discovery and interpretation using this exceptional and freely available dataset.

My group and others previously developed analysis strategies for GWAS with metabolomics data,[7,8] a field that is similar in many ways to that of pQTL studies. In particular, we showed that partial correlations between metabolites can reconstruct metabolic networks[9,10] and that the hypothesis-free testing of all of the ratios between metabolites can substantially strengthen the association signals, in several cases elevating genetic loci out of the background noise.[11,12] Both approaches are related in that they identify biological relationships between individual molecules through their shared genetic and nongenetic variance, which can then be integrated into larger metabolic networks, such as the atlas of genetic influences of the human metabolome[13] and more recent versions thereof.[14] Previous GWAS with proteomics suggest that Gaussian graphical models (GGMs) built from partial correlations and ratios between protein

This is an open access article under the CC BY license (http://creativecommons.org/licenses/by/4.0/).

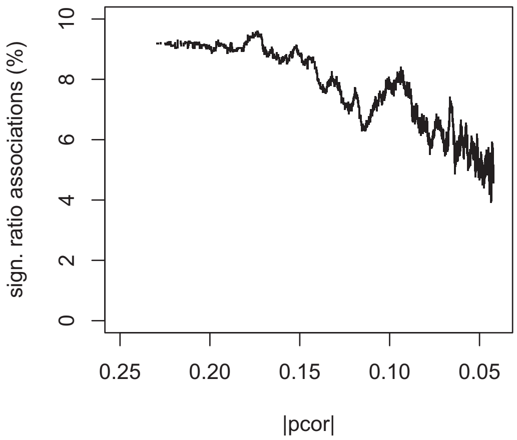

Figure 1. Percentage of Bonferroni significant protein ratio pair associations (p-gain >10 × 179,923) as a function of the partial correlation |pcor| between the protein pair

A moving average with a window size of 10,000 data points was used.

levels can reveal biologically relevant protein-protein interactions,[5] but the approach has never been tested at scale.

This article hypothesizes that a GWAS with ratios between protein levels can identify associations and novel links between protein pairs that have not been identified using current GWAS approaches. However, the computational costs of conducting a full-fledged all-against-all ratio GWAS are prohibitive at this point, estimated to several hundred thousand pounds Sterling on the DNAnexus AWS-based platform for a single run of a full-fledged all-against-all ratio GWAS, not considering costs associated with the handling of the generated data. This challenge will be aggravated in the future by the expected increases in proteome coverage.

This article, therefore, takes a more economic approach and tests genetic associations with ratios between proteins that are partially correlated and therefore more likely to be related through some biological process.[15] For each pQTL reported by the UKB PPP consortium that implicated one of two partially correlated proteins, the ratio between the levels of these two proteins is tested for association with the pQTL variant. Then, a GWAS is conducted on those ratios that increased the strength of association at an already known pQTL locus (see the flowchart of this study in Figure S1). It will be shown in the following that by using this approach pQTLs could be identified that were not discovered by the standard GWAS with protein levels conducted by the UKB PPP consortium,[6] and furthermore, that genetic associations with ratios can uncover biologically relevant links between two or more proteins based on their shared genetic and nongenetic variance. Selected cases of biomedical interest are discussed and an interpretation of why I believe ratios work is provided.

RESULTS

Identification of ratio QTLs (rQTLs) at established pQTL loci

The increase in the strength of an association with ratios is quantified by the p-gain, which is defined as the smaller of the two p values for the single-protein associations divided by that for the ratio association.[11] A p-gain of 10 is the equivalent of a nominal p value for a single test; in other words, a p-gain of 10 is expected to be observed by chance in 5% of the cases when ratios between two random proteins are tested. The following requires Bonferroni levels of significance for p values and p-gains throughout and refers to protein ratio associations with significant p-gains as rQTLs.

Quality control (QC) of the Olink data was performed by the UKB PPP before the data was shared with the user. These QC steps include outlier removal and removal of samples of low quality. The number of data points that passed QC was above 97.6% for all Olink panels (see method details). We split the UKB cohort into a discovery set comprising 43,000 individuals and a replication set of 8,700 individuals based on the data field "genetic ethnic grouping" being equal/not equal to "Caucasian" (see UKB documentation on data field 22006) and further limit the analysis to samples collected at baseline.

A total of 179,923 ratio-variant pairs were tested for association, selected as the overlap of 11,936 Bonferroni-significant GGM edges ($p < 4.7 \times 10^{-8}$ or $|pcor| > 1.76 \times 10^{-3}$; Table S2) and 10,248 Bonferroni-significant pQTLs ($p < 3.4 \times 10^{-11}$; Table S3) from the UKB PPP GWAS.[6] A total of 10,760 ratio associations (5.98%) had a Bonferroni-significant p-gain (>10* 179,923), and of these 4,248 (41.4%) replicated in the genetically "non-Caucasian" cohort (p-gain >10 × 10,760). The 4,248 replicated ratio associations covered 2,821 unique protein pairs between 1,001 of the 1,463 (68.4%) proteins assayed on the Olink platform and 926 of the 5,717 (16.2%) genetic variants reported as pQTL variants by the UKB PPP GWAS (Tables S4 and S5). The likelihood of finding a significant ratio association for a protein pair increased with the strength of their partial correlation from ~5% for uncorrelated proteins to 9% for |pcor| ~0.2 (Figure 1), supporting our choice to prioritize GGM protein pairs.

A selection of pharmaceutically relevant rQTLs is provided in Table 1, including associations with a ratio between a *cis*- and a *trans*-located protein, where the *cis*-protein is the target of an approved drug (*Tclin* according to Pharos[16]).

Identification of rQTLs in a GWAS with ratios

A GWAS was conducted on the 2,821 ratios using the genotyped UKB data. For each ratio, I retained the strongest associations that reached a Bonferroni level of significance of p value $<5 \times 10^{-8}/2{,}821$, a p-gain $>10^7 \times 2{,}821$, and that were more distant than 1 million bp from any other significant association with the same ratio. I identified 8,462 ratio-variant pairs with 2,095 unique variants that satisfied this criterion, which corresponds to a discovery per tested ratio of on average three independent GWAS signals with a significant p-gain (Figures 2 and S2; Table S6). The ratios with the largest number of rQTLs discovered were heparin-binding EGF-like growth factor (HBEGF) divided by platelet-derived growth factor subunit A (PDGFA) (N = 25) and integrin subunit β1 binding protein 2 (ITGB1BP2) divided by microtubule interacting and transport, domain containing 1 (MITD1) (N = 24). A total of 999 proteins were implicated in at least one rQTL, with a median of eight rQTLs per protein. The two most frequently occurring proteins were ITGB1BP2, with 259 rQTLs, and ectodysplasin A receptor, with 237 rQTLs. A total of 2,527 (29.9%) of the 8,462 rQTLs were more distant than 10^6 bp

Table 1. rQTLs that implicate drug targets in the *cis*-position

cis-Protein	Ratio	Chr	Pos	rsID	−logP.1	−logP.2	−logP.3	logPgain	GWAS traits
CA6	CA6/DNER	1	9,034,598	rs3765963	2,878.1	0.6	3,235.0	356.9	—
LEPR	IL6ST/LEPR	1	66,073,982	rs10399687	0.1	225.1	270.6	45.5	Blood cell traits
SLAMF7	ICAM3/SLAMF7	1	160,720,074	rs11581248	3.7	2,094.1	2,631.3	537.2	—
NECTIN4	NBL1/NECTIN4	1	161,049,509	rs35434391	0.4	396.6	643.6	247.0	Blood cell traits
SELP	SELP/VSIR	1	169,563,951	rs6136	349.1	0.3	663.3	314.2	Activated partial thromboplastin time
DPP4	DPP4/ITGB1	2	162,930,725	rs13015258	229.0	0.9	317.5	88.5	—
PDCD1	PDCD1/TNFRSF8	2	242,801,752	–	271.2	0.3	364.4	93.1	—
CD38	CD38/RELT	4	15,775,851	rs28703311	894.1	0.9	1,030.5	136.4	—
PDGFRA	IL6ST/PDGFRA	4	55,139,771	rs35597368	0.6	364.8	439.3	74.4	Impedance of the legs, peak expiratory flow
F2R	DAG1/F2R	5	76,028,124	rs168753	0.4	56.1	276.6	220.5	—
FLT4	FLT4/ICAM2	5	180,057,293	rs34221241	389.3	0.6	516.6	127.4	—
IGF2R	CTSO/IGF2R	6	160,409,894	rs75474551	106.3	366.8	434.3	67.5	—
AKR1B1	AKR1B1/SUGT1	7	134,135,621	rs2229542	92.6	0.4	172.2	79.5	—
IMPA1	IMPA1/TBCC	8	82,583,771	rs1967328	176.4	0.9	329.5	153.0	—
CA1	CA1/HMBS	8	86,256,210	rs12544332	35.7	0.5	114.2	78.5	Blood cell traits
CA3	CA1/CA3	8	86,351,051	rs2072696	6.7	109.1	387.6	278.6	—
CD274	CD274/EFNA4	9	5,453,260	rs822340	446.6	0.6	500.4	53.8	Ulcerative colitis
IL2RA	IL-2RA/TNFRSF4	10	6,095,928	rs12722497	1,440.8	0.1	1,892.6	451.8	Streptococcal throat infections
LAG3	LAG3/VCAM1	12	6,885,076	rs3782735	137.5	0.6	169.1	31.6	—
TXNRD1	NUDT5/TXNRD1	12	104,707,047	rs201402862	0.4	20.3	41.8	21.5	—
FLT3	FLT3/FLT3LG	13	28,637,838	rs9554228	34.2	36.0	53.9	17.8	Age at menarche, blood cell traits, body size, mass and fat traits, rheumatoid arthritis
IL-4R	CKAP4/IL-4R	16	27,327,214	rs8060025	0.3	449.7	497.6	48.0	Asthma, eosinophils
CA5A	AGXT/CA5A	16	87,927,222	rs55870502	0.9	2,387.8	3,738.1	1,350.3	Basophils
COMT	COMT/HNRNPK	22	19,951,271	rs4680	1,008.4	0.7	3,043.4	2,035.1	Basal metabolic rate, systolic blood pressure, body fat traits

Selected rQTLs that include a ratio between a *cis*- and a *trans*-located protein, with the *cis*-protein being the target of an approved drug (*Tclin* according to Pharos[16]). The negative $\log_{10}$-transformed p values for the association with the single proteins (−logP.1, −logP.2) and the ratio (−logP.3) and the $\log_{10}$-transformed p-gain (logPgain) for the rQTL are reported; the GWAS traits were annotated using PhenoScanner[17] (details in Table S4).

from any pQTL reported by the UKB PPP GWAS for one of the two proteins in the respective ratio and thus represent previously nonreported pQTLs, which corresponds to an increase of 24.7% in genetic signals derived from the UKB PPP Olink data using ratios compared to the standard approach.

To investigate whether these rQTLs provided new insights of biomedical interest, I annotated the 2,095 rQTL variants identified in this study and the 5,717 pQTL variants reported by the UKB PPP GWAS using PhenoScanner[17] for association with 446 distinct GWAS traits (Tables S6 and S3, respectively). I identified 322 rQTL variants that were more distant than 10^6 bp from any pQTL variant on the same GWAS trait, implicating 874 rQTLs in a total of 4,700 coassociations with GWAS traits (Table S7).

These rQTLs provide new evidence to support drug target selection. For instance, rs3764640 associated with the ratio STK11/USP8 ($-\log_{10}(p) = 13.8$, $\log_{10}(\text{p-gain}) = 10.5$). The variant is an intragenic SNP in the *STK11* gene and associated with the presence versus absence of psychosis in Alzheimer disease (AD) cases.[18] STK11 is a serine/threonine-protein kinase, and USP8 may play a role in the degradation of activated protein kinases by ubiquitination,[19] which would explain the significant p-gain for the ratio. This rQTL hence not only supports a role of STK11 in AD pathology but it also provides further insights into the putative underlying biological pathways, suggesting that medicinal modification of STK11 or its phosphorylation targets may affect the AD-related phenotypes.

A second example is a region of high linkage disequilibrium (LD) on chromosome 5 (Figure 3), which is a major risk locus for inflammatory bowel disease (IBD).[20] The most likely causal gene prioritized by multiple GWAS, based on its function and the presence of

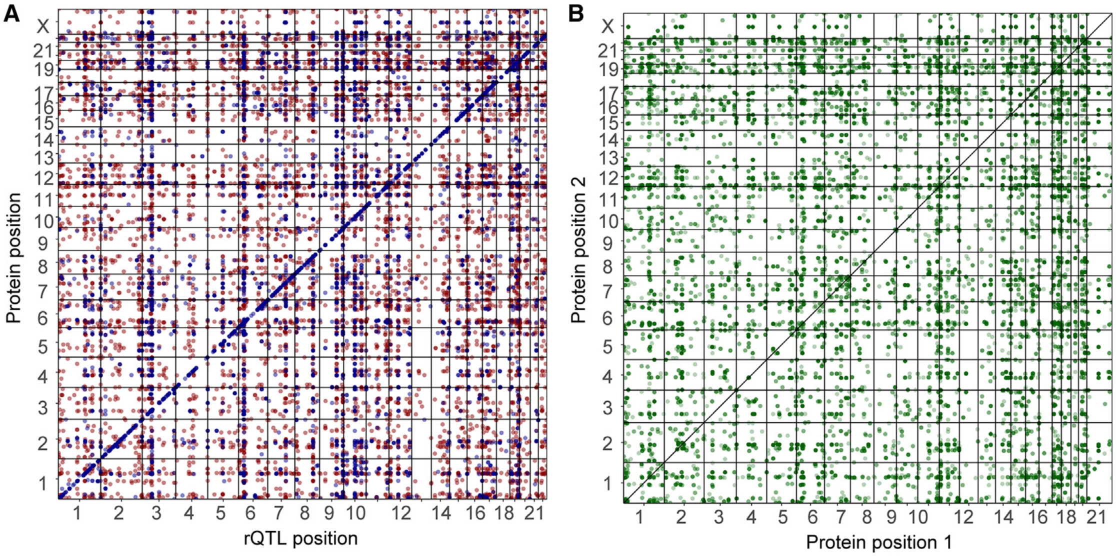

Figure 2. Two-dimensional Manhattan plots

(A) The position of the rQTL plotted against the position of the genes coding for the two proteins in the ratio, the stronger of the two single protein associations is in blue, the weaker in red.

(B) The positions of the genes coding for the two proteins in an rQTL ratio plotted against each other; darker colors indicate multiple rQTLs with the same ratio.

an amino acid-changing variant, was *macrophage stimulating 1 (MST1)*. However, this view has been challenged, proposing *glutathione peroxidase 1* (*GPX1*) as a causal gene instead, supported by biochemical experiments showing that a cosegregating amino acid-changing variant in *GPX1* reduced the activity of this antioxidant enzyme.[21] Here, 14 ratios between 16 proteins were identified that associated with a significant p-gain at this locus (Table S8). Seven were ratios of pyruvate kinase, liver, and red blood cells (*PKLR*) with proteins involved in hemoglobin metabolism, including hydroxyacylglutathione hydrolase (HAGH), hydroxymethylbilane synthase (HMBS), arginase 1, and biliverdin reductase B. The biochemical properties of these proteins clearly support a causal role for GPX1 in an oxidative stress-related phenotype, likely related to hemoglobin metabolism in red blood cells. However, four of the ratios were with the *cis*-encoded protein dystroglycan 1 (DAG1) and several proteins not related to red blood cell metabolism, suggesting the presence of a second, likely independent, causal gene at this locus, which would cosegregate with the *GPX1* variant due to the high LD in this region. Whether both pathways are driving factors of the IBD association requires further investigation. Important for the present study is that this case exemplifies the kind of insights that can be drawn from using rQTLs and their value for drug target evaluation and hypothesis generation.

Discovery of *cis*-pQTLs

Observation of *cis*-pQTLs is considered genetic evidence to confirm the target specificity of the respective affinity binding assay. Sun et al.[6] found a *cis*-pQTL for 1,163 (79.5%) of the 1,463 assayed proteins. Here, I report 39 additional genetic variants that associated with a ratio that involves a protein located in-*cis* and a second protein located in-*trans* (Table 2). These *cis*-pQTLs became presumably discoverable because the *trans*-proteins in the ratios captured some unidentified shared nongenetic variance, accounting for which led to the significant p-gains. The corresponding 39 proteins include three Olink targets for which no genetic signal had been found in the UKB PPP GWAS at all (ARHGEF12, EIF4EBP1, INPPL1) and thus provide genetic evidence that the respective antibodies bind their designated targets. Even by using only a subset of all of the possible ratios, I identified 13% of the 300 *cis*-pQTLs that were not accounted for so far, increasing confidence in the target specificity of the Olink platform for these proteins. More may be identified in an all-against-all ratio approach.

Refinement of the rQTL loci

For economic reasons, the GWAS was conducted using the genotyped variants only and therefore I may have missed variants of interest. For each of the 8,462 rQTLs, I therefore refined the associations within ±500,000 bp of the respective lead variant by using the imputed UKB genotype data, both in the discovery and in the replication cohort. The summary statistics are provided for all 8,462 refined regions on FigShare (Data S1, https://doi.org/10.6084/m9.figshare.23695398). These data can also be used to further refine the loci of interest—for instance, to identify potentially multiple independent signals using SuSiE[23] or to test for colocalization with other traits of interest using coloc.[24] To visualize individual rQTLs regional association plots were generated for all rQTLs, both in the discovery and the replication cohort (Figure S3).

I then used coloc[24] to ask whether the two proteins in a ratio shared the same genetic signal (Q.12), whether any of the two proteins shared a signal with the ratio (Q.13 and Q.23), and whether the signal for the ratio was shared between discovery and the replication cohort (Q.33.repli). Table S6 provides the most likely hypothesis for each of these four questions, together with its posterior probability. In 7,414 (87.6%) of the 8,462 cases, at least one of

CellPress
OPEN ACCESS

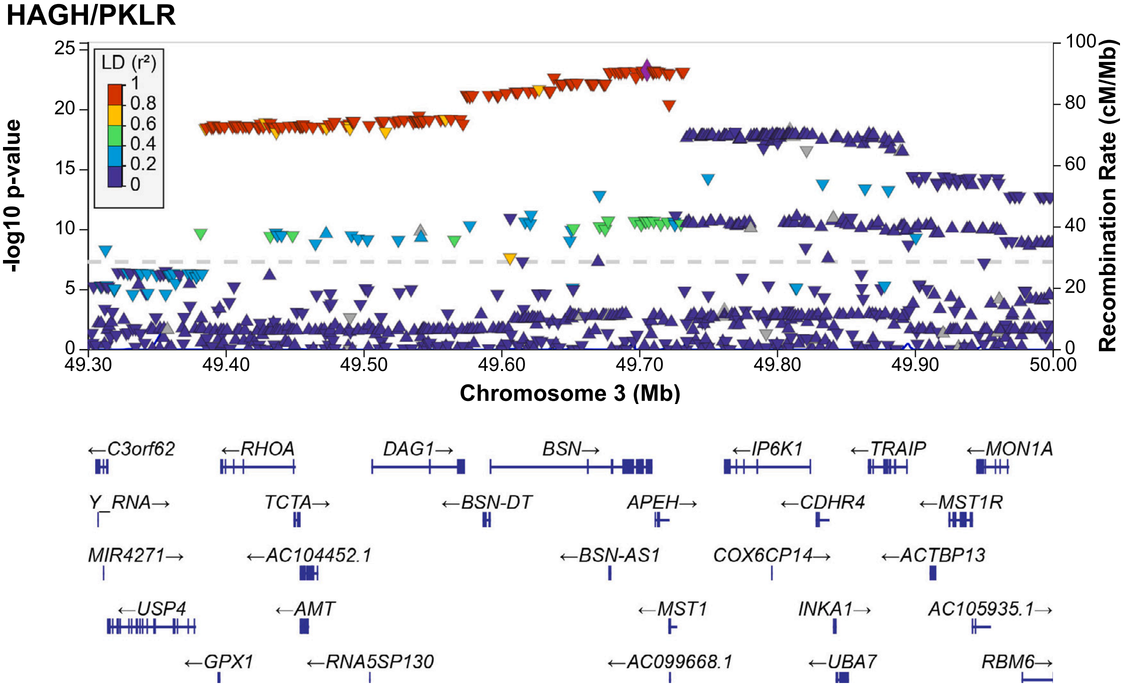

Figure 3. Regional association plot for the association of the HAGH/PKLR ratio at a major IBD locus on Chr3
Plot created using LocusZoom.[22]

the proteins shared a genetic signal with the ratio (Q.13 = H4 or Q.23 = H4), in 1,305 (15.4%) cases both proteins shared a signal with the ratio (Q.13 = H4 and Q.23 = H4), and in 489 (5.8%) cases there was no signal detectable for either of the two proteins alone (Q.13 = H2 and Q.23 = H2 and Q.12 = H0). A total of 6,775 of the 8,462 rQTLs (80.1%) shared a genetic signal between discovery and the replication cohort (Q.33.repli = H4).

For each rQTL region, the variant with the strongest association with the ratio in the discovery cohort was designated as the lead variant, and I asked whether the association on this variant replicated. Requiring in the discovery cohort $p < 5 \times 10^{-8}/2{,}821$ and p-gain $>10 \times 10^{6} \times 2{,}821$ and in the replication cohort $p < 0.05/8{,}462$ and p-gain $>10 \times 8{,}462$, I identified 4,181 rQTLs (49.4%) that satisfied this stringent Bonferroni significance criterion. Considering that 80.1% of the rQTLs shared the same genetic signal between discovery and the replication cohort, it is likely that more rQTLs can be replicated when more samples become available.

Why do ratios work and what do they represent?

With P_1 and P_2 representing the levels of two blood circulating proteins (the indices of the individual samples were suppressed) two linear models can be fit to the log-scaled protein levels by selecting parameters α_i, β_i, and γ_i such that they minimize the square of the nonexplained variance ε_i in the following equation:

$$\log(P_i) = \alpha_i + \beta_i \times \text{SNP} + \gamma_i \times W + \varepsilon_i \quad \text{for } i \in \{1,2\}$$

SNP represents the number of effect alleles (0, 1, 2) of a given genetic variant in a given sample and W denotes some nonidentified nongenetic variance that is shared by both proteins. Using the identity log(A/B) = log(A) − log(B), the ratio can then be written as:

$$\log(P_1 / P_2) = (\alpha_1 - \alpha_2) + (\beta_1 - \beta_2) \times \text{SNP} + (\gamma_1 - \gamma_2) \times W + (\varepsilon_1 - \varepsilon_2).$$

Because the significance level (p-value) of the association with the variant depends on the proportion of the variance that is explained by the genetic term (SNP) compared to the remaining variance ($W + \varepsilon$), the strength of the association with the ratio can increase under two conditions: (1) when β_1 and β_2 have opposite signs, or (2) when γ_1 and γ_2 are of comparable size and nonzero.

β_1 and β_2 having opposite signs implies that the genetic variant increases the levels of one protein while decreasing those of the other (Figure 4). When working with metabolites, this situation can occur when the ratio represents a substrate-product pair of an enzyme whose efficacy is affected by the genetic variant. Many such cases have been reported.[12,13,25] For proteins, a possible scenario is a genetic variant that increases the expression of one protein that acts as a suppressor of a second protein. One example is the association of rs1065853, which is in LD with coding SNP rs7412 in *APOE*, and the ratio between low-density lipoprotein receptor (LDLR) and proprotein convertase subtilisin/kexin type-9 (PCSK9) protein levels ($\log_{10}$(p-gain) = 67.2). PCSK9 binds LDLR and targets it for degradation.[26] The availability of PCSK9 to degrade LDLR in turn is limited by binding to apolipoprotein B,[27] the levels of which are associated with rs7412 in *APOE*.

Table 2. Novel *cis*-pQTLs

cis-Protein	Ratio	Chr	Pos	rsID	−logP.1	−logP.2	−logP.3	logPgain
MNDA	MNDA/NCF2	1	158,788,542	rs2875712	5.7	0.3	16.9	11.2
EPCAM	EPCAM/GPA33	2	47,773,540	rs6708696	1.6	1.3	15.5	13.9
ANXA4	ANXA4/LACTB2	2	70,033,584	rs2228203	11.6	3.8	232.7	221.1
TMSB10	DBI/TMSB10	2	85,133,861	rs13409738	0.0	8.1	36.8	28.7
NCK2	NCK2/PLA2G4A	2	106,452,253	rs10169998	4.6	0.0	36.4	31.8
TGFBR2	TGFBR2/TNFRSF1A	3	30,729,510	rs114836705	8.2	0.4	25.0	16.9
PPP1R2	PPP1R2/SNAP23	3	195,238,559	rs34950021	1.9	0.8	26.6	24.7
CXCL3	CXCL3/CXCL5	4	74,797,139	rs352024	0.6	514.2	1,229.3	715.1
FYB1	FYB1/PPP1R12A	5	39,338,358	rs3822462	1.8	0.7	14.1	12.3
DAB2	DAB2/NCK2	5	39,427,481	rs75839063	2.5	0.0	20.3	17.8
HBEGF	HBEGF/PDGFA	5	139,720,400	rs2237077	7.6	0.8	31.1	23.4
PDLIM7	PDLIM7/SRC	5	176,922,643	rs335428	7.5	1.0	28.9	21.4
MPIG6B	MPIG6B/PLXNA4	6	31,346,193	rs2507982	0.3	5.1	18.5	13.5
MAP3K5	MAP3K5/MAVS	6	136,888,889	rs56379668	4.0	1.9	31.7	27.7
VTA1	RWDD1/VTA1	6	142,641,606	rs12189801	0.6	4.2	21.1	16.9
LAT2	CLIP2/LAT2	7	73,780,812	rs512023	4.9	4.8	59.6	54.7
CASP2	CASP2/NUB1	7	142,986,684	rs3181165	2.8	0.5	14.3	11.4
PLPBP	PLPBP/RWDD1	8	37,635,649	rs7463174	8.7	1.1	37.9	29.2
EIF4EBP1[a]	DNPH1/EIF4EBP1	8	37,884,310	rs28797500	0.5	4.3	18.4	14.1
LYN	KIFBP/LYN	8	56,785,133	rs6985703	0.2	5.5	18.5	12.9
INPPL1[a]	BANK1/INPPL1	11	72,064,041	rs79658353	0.1	1.7	13.0	11.3
PPME1	ATG4A/PPME1	11	73,948,875	rs79153613	0.6	9.5	30.1	20.6
ARHGEF12[a]	ARHGEF12/AXIN1	11	120,278,477	rs34172482	4.6	0.1	37.7	33.1
IRAG2	CRACR2A/IRAG2	12	25,243,115	rs1908946	0.3	3.5	16.4	12.8
METAP2	EIF4B/METAP2	12	95,830,338	rs159853	0.6	8.9	27.4	18.5
TRIAP1	TMSB10/TRIAP1	12	120,902,007	rs542407	0.3	3.6	14.7	11.1
SRP14	APEX1/SRP14	15	40,325,829	rs6492926	0.0	10.9	25.3	14.4
SNAP23	CD69/SNAP23	15	42,808,309	rs73404730	0.8	1.7	21.2	19.6
PPIB	MANF/PPIB	15	64,780,971	rs73452261	1.0	1.9	29.2	27.3
MESD	MANF/MESD	15	81,279,706	rs57967327	0.3	5.4	74.5	69.1
CORO1A	CORO1A/TBC1D5	16	30,147,265	rs7201780	4.9	0.0	18.2	13.3
STX4	DNMBP/STX4	16	31,004,812	rs12445568	0.3	6.7	20.5	13.9
AHSP	AHSP/CA2	16	31,463,216	rs4889659	9.9	0.0	26.3	16.4
VPS53	SCAMP3/VPS53	17	400,933	rs9916346	0.3	2.9	14.7	11.8
STK11	STK11/USP8	19	1,207,238	rs3764640	3.3	0.6	13.8	10.5
CDKN2D	CDKN2D/TACC3	19	9,868,278	rs10420364	0.5	6.7	45.5	38.7
CDC37	CDC37/PLA2G4A	19	10,523,086	rs10854116	4.4	0.8	47.0	42.6
TBCB	MITD1/TBCB	19	36,593,915	rs61741470	0.3	9.7	130.7	121.0
CRKL	CRKL/DBNL	22	21,139,239	rs117858197	1.6	0.5	34.8	33.1

List of 39 genetic variants that associated with a ratio that involves a protein located less than 1 MB from the variant (*cis*-protein) and that has no *cis*-pQTL in the UKB PPP GWAS. The negative $\log_{10}$-transformed p values for the association with the single proteins (−logP.1, −logP.2) and the ratio (−logP.3) and the $\log_{10}$-transformed p-gain (logPgain) for the rQTL are reported (details in Table S6).
[a]This protein has no pQTL in the UKB PPP GWAS.

If only one of the proteins is affected by the genetic variant, then observing a significant p-gain implies that γ_1 and γ_2 must be of comparable size and nonzero, and the association with the ratio indicates the presence of some nongenetic variance that is shared by both proteins. For instance, ITGB1BP2 has only five *trans*-pQTLs in the UKB PPP GWAS of moderate effect size, but occurs with 26 different ratios in 259 rQTLs in the present study's GWAS, the strongest with a log10(p-gain) = 1,115.5 for the association of rs4680 with the ratio catechol-*O*-methyltransferase (COMT) divided by ITGB1BP2 at the *COMT* gene locus. The association of ITGB1BP2 with rs4680 was not significant (p = 0.69). The ratio with the largest number of rQTLs

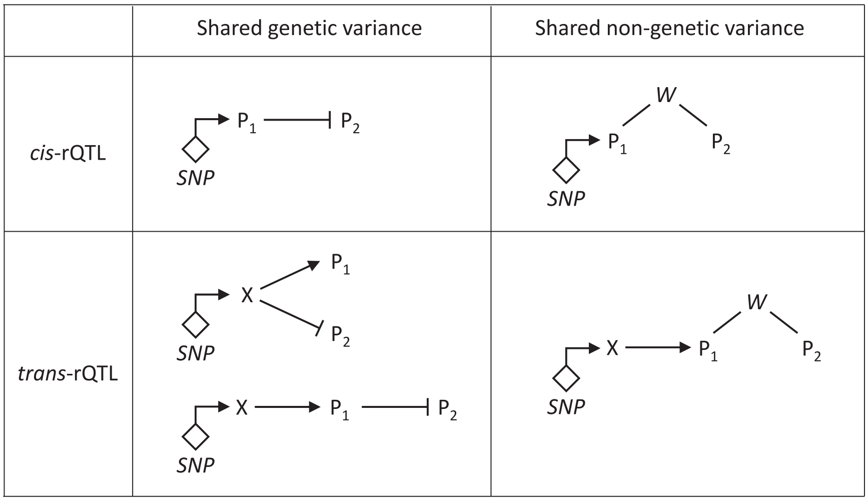

Figure 4. Possible scenarios that can lead to a significant p-gain in a ratio association

P1 and P2 are the proteins in the ratio that associates with the genetic variant *SNP*, X is the causal *cis*-encoded protein in the case of a *trans*-rQTLs, and *W* denotes some unidentified shared nongenetic variance.

in the GWAS was ITGB1BP2/MITD1, which had 24 rQTLs compared to only 2 pQTLs for MITD1 in the UKB PPP GWAS (Figure 5). Intriguingly, both proteins are highly correlated (Pearson $r^2 = 0.86$), suggesting that their correlation is driven by some shared but not identified factor. The strongest correlations with one of the clinical biochemistry and blood traits available in UKB was with platelet count ($r^2 = 0.12$ with ITGB1BP2, $r^2 = 0.10$ with MITD1, and $r^2 = 0.025$ with the ratio), which are too weak to explain the full correlation between both proteins, suggesting that a driving factor for this association is related to some more specific, probably blood cell-type-related trait that is not readily available in the UKB phenotype dataset.

There are multiple possible causes that can lead to a significant p-gain in a ratio association, as schematized in Figure 4; some rQTLs reveal the presence of shared genetic variance, whereas others suggest the proteins in the ratio being linked through some shared nongenetic processes. It is interesting to note in this context that adjusting for hidden factors by using genomic or proteomic surrogates can also lead to a substantial improvement in pQTL detection.[28]

What can be learned from rQTLs?

To evaluate the enrichment in protein pairs that were linked through significant ratio associations and/or GGM edges, the STRING database of protein-protein interactions was used (Table S5). Of the 2,281 protein pairs, 168 pairs (6.0%) had a protein-protein interaction link in the STRING database with a high confidence score (>0.7), and random pairs between these proteins had on average only 22.1 links (SD = 4.2, based on 100 samplings), which corresponds to a 7.6-fold enrichment. For comparison, of the 11,936 protein pairs linked through significant GGM edges, 465 pairs (3.9%) had a protein-protein interaction reported in STRING, whereas random sampling yielded an average of 89.4 (SD = 9.4), which corresponds to a 5.2-fold enrichment for proteins linked through GGM edges alone.

Using cytokine and cytokine-receptor annotations from CytokineLink,[29] 39 protein pairs were identified with an rQTL that involved cytokine-cytokine pairs, 15 receptor-receptor pairs, and 9 cytokine-receptor pairs, 7 of which were known and 2 were new (colony-stimulating factor 1:lymphotoxin β receptor and C-X-C motif chemokine ligand 9 [CXCL9]:tumor necrosis factor receptor superfamily member 9 [TNFRSF9]). CytokineLink predicted 1,542 cytokine-cytokine interactions between 77 cytokines from the Olink platform (out of 77 × 76/2 = 2,926 possible interactions). The number of cytokine pairs that were involved in rQTLs was significantly enriched (30 out of 39 compared to 1,512 out of 2,887 with no rQTL, $p < 0.002$, Fisher exact test).

When multiple proteins associate in rQTLs with the same variant, networks of related proteins can be constructed. Here, an example is presented of how a single pQTL around a pharmaceutically interesting protein can be extended into a network of potentially interacting proteins. NFATC1 (nuclear factor of activated T cells 1) is a key transcription factor and regulator of the immune response[30] and a molecular target for immunosuppressive drugs such as cyclosporin A.[31] NFATC1 has been implicated in the pathogenesis and targeted therapy of hematological malignancies.[32,33] rs657693 is a *cis*-pQTL for NFATC1 in the UKB PPP GWAS and one of only two genetic associations for this protein. rs657693 is identified here as an rQTL for the ratio of NFATC1 with 16 other proteins (AXIN1, BACH1, BANK1, BCR, CASP2, CD69, EIF4G1, FADD, FOXO1, IKBKG, INPPL1, IRAK1, LBR, PTPN6, SPRY2, TJAP1; Table S4), none of which had a significant association with this variant alone. Of the 16 proteins 9 had a second replicated rQTL in a ratio with NFATC1 elsewhere in the genome, and in all of these cases, the other protein in the ratio was the driving pQTL, with 5 of them being *cis*-pQTLs (AXIN1, BANK1, FOXO1, SPRY2, TJAP1). GWAS identified additional rQTLs, including *cis*-rQTLs for BCR (rs713617), cluster of differentiation 69 (CD69) (rs7309767), and *Fas*-associated protein with death domain (FADD) (rs7939734). Ingenuity Pathway Analysis identified a number of functional links between these proteins, generating a network of proteins that can now be linked through genetic evidence and rQTLs to the NFATC1 locus, potentially supporting the development of new immunosuppressive drugs (Figure S4). For instance, it has been shown that silencing NFATC1 results in the phosphorylation of forkhead box O1 (FOXO1) and thereby plays a role in cell differentiation.[34] Expression of NFATC1 and nine of the proteins in its ratio (AXIN1, BCR, CASP2, CD69, EIF4G1, FADD, IRAK1, LBR, PTPN6) is enriched in leukemia cells (false discovery rate = 0.003).[35] Interleukin 1 receptor associated kinase 1 (IRAK1) is an emerging therapeutic target in hematologic malignancies, and it has been suggested that IRAKs participate in regulatory interactions with FADD.[36] Indeed, FADD has

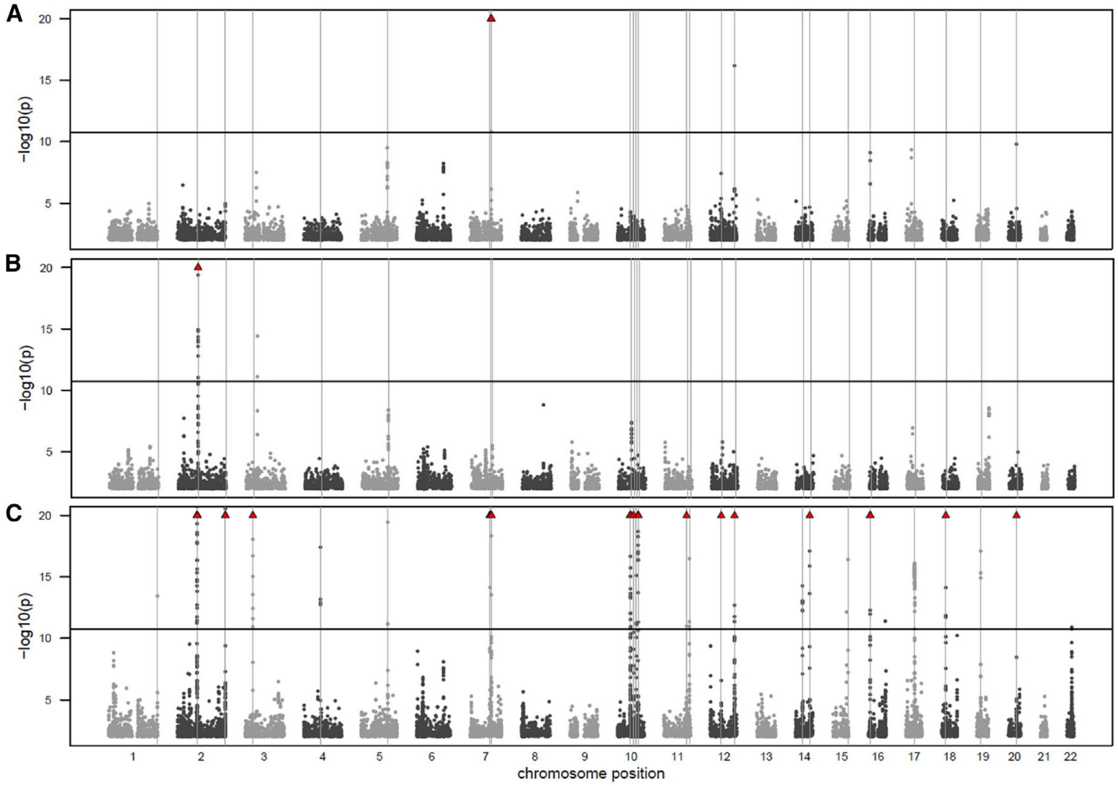

Figure 5. Example of a ratio that leads to the discovery of novel signals

(A–C) Manhattan plots for the GWAS with (A) ITGB1BP2, (B) MITD1, and (C) the ratio ITGB1BP2/MITD1. Associations with p values exceeding 10^{-20} are indicated by red triangles. Vertical lines indicate 24 Bonferroni-significant ($p < 5 \times 10^{-8}/2821$) rQTLs for the ratio. Manhattan plots for all 2,821 ratio GWAS are available as online (Figure S1 and Data S1, https://doi.org/10.6084/m9.figshare.23695398).

been shown to physically interact with IRAK1,[37] lending further experimental support to this rQTL-derived network and its role in T cell development.

To further explore the benefits of all-against-all ratios, the associations of SNP rs12075 were computed with all possible ratios between 76 cytokines that are in the Olink panel. rs12075 (1:159175354:G:A) is an amino acid changing variant (c.125G>A, p.Gly42Asp) in the *atypical chemokine receptor 1* gene (*ACKR1* aka *DARC*). The glycine variant defines the Fy^a allele and the aspartate variant defines the Fy^b allele of the Duffy blood group system.[38] DARC is clinically important because it is the entry point for the human malaria parasite *Plasmodium vivax*. Individuals with two copies of the Fy^a allele or a silenced Fy^b allele are resistant to *P. vivax* infection. A structural basis for DARC binding to *P. vivax*'s Duffy-binding protein involving the region around the p.Gly42Asp variant has been proposed.[39]

Eleven cytokines are associated in the UKB PPP GWAS at the *ACKR1* locus (CCL2 [C-C motif chemokine ligand 2], CCL7, CCL8, CCL11, CCL13, CCL14, CCL17, CCL26, CXCL1, CXCL6, CXCL8; Table S3). CCL7 and CCL8 protein levels increased with the copy number of the Fy^a allele, whereas levels of the other nine cytokines decreased with that variant. DARC controls chemokine levels through promiscuous binding.[40] The associations with these cytokines thus match the function of DARC. In the discovery study, rs12075 associated with three ratios (CCL13/CCL8, CCL2/CCL7, and CCL11/CCL7; Table S4), and the strongest association of rs12075 was with the ratio between CCL8 and CCL13. An interaction between these two cytokines has been experimentally established by bidirectional immunoligand blotting.[41] Testing the association of rs12075 with all possible ratios between the 76 cytokines on the Olink panel implicated 12 additional cytokines (CCL3, CCL4, CCL14, CXCL11, CXCL12, HGF, IL-7, PDGFA, TGFB1, THPO, TNFSF13, TNFSF14) in significant (p-gain > 10^{10}) rQTLs (Figure S5; Table S9). It goes beyond the scope of the present study to interpret these associations in further detail. The takeaway message here is that using ratios not only identified additional cytokines that associate with the Duffy blood type but they also suggest interactions between specific pairs of proteins, such as CXCL6, which occurs in a significant ratio with CCL8, but not with CCL7. Especially at pleiotropic loci, where multiple proteins associate with a clinically relevant variant, it may be worthwhile to conduct this kind of all-against-all ratio analysis, using a subset of functionally related protein, as done in this example, or even extending the ratio analysis to the full protein panel.

DISCUSSION

In this study, a GWAS at scale is presented with ratios between blood circulating protein levels, using the recently released Olink

proteomics data for almost 1,500 proteins measured in the blood plasma of 54,000 participants of the UKB. Using ratios, increases in the strengths of association by up to several hundred orders of magnitude were observed, involving two-thirds of the proteins targeted by the Olink platform, increasing the strength of association at 16% of the pQTLs from the UKB PPP GWAS. Novel *cis*-pQTLs were reported for 13% of the 300 Olink proteins for which such a target-confirmatory QTL had not been identified so far and uncovered over 2,500 novel QTLs with ratios at loci that had not been highlighted by the UKB PPP GWAS using single protein levels, which corresponds to a 25% increase in the discovery rate.

I argue that ratios can account for unidentified genetic and/or nongenetic variance that is shared between the associated protein pairs (Figure 4). rQTL protein pairs were 7.2-fold enriched in known protein-protein interactions, demonstrating that they add substantial new information to hypothesis generation and providing a broad set of protein-protein relationships that can be mined using network pharmacology (the NFATC1 example)[42] and systems immunology (the ACKR1 example)[43,44] approaches. I further reported selected examples that illustrate insights gained from using ratios, adding new information to established loci (the GPX1 with IBD example) and identifying entirely novel loci (the STK11 with AD example).

Because I could only discuss a fraction of the biologically relevant findings in this paper, I freely share the full summary statistics of the GWAS using array-genotyped data and of the refinement using imputed genotype data, together with the corresponding Manhattan and regional association plots. These data represent a rich resource for biomedical hypothesis generation that complements the data generated by the UKB PPP GWAS and should be of particular value for pharmaceutical drug target development.

I hope to have demonstrated the benefits of analyzing ratios between protein levels at scale, an approach that I believe has already shown its benefits in the metabolomics field. Future work is needed to further speed up the calculation of ratio associations, especially in light of the broadening proteomics panels and their increased application in large-scale cohorts. Also, theoretical development and generalization of the concept of ratios to more general classes of multivariate association tests may further improve the power for the detection of genetic signals.

Limitations of the study

The following caveats apply: I analyzed only a subset of all of the possible ratios. Although the likelihood of finding a significant ratio association for a protein pair increased with the strength of their partial correlation, even for uncorrelated proteins, this likelihood remains high (5%), supporting the testing of all possible ratios in future GWAS, if resources permit. Indeed, I hope that the present study motivates further methods development that could render all-against-all ratio testing computationally feasible, and perhaps also more research into formal statistical methods that may generalize the analysis of combinations of quantitative traits as dependent variables in GWAS.

As for all GWAS, this study is hypothesis generating by nature. An rQTL suggests a potential relationship between the two proteins in the ratios, plus potentially a third protein encoded at the genetic locus in the case of *trans*-rQTLs. These relationships can be of different natures, ranging from a direct physical interaction over indirect regulatory interactions to broad shared nongenetic factors. Also, proteins may have multiple functions and can be associated with multiple pathways, which needs to be considered in the interpretation of the rQTLs on a one-by-one basis. I also acknowledge that there is presently no established causal inference framework, which somewhat limits the interpretation of the findings from a Mendelian randomization perspective.

It should also be noted that affinity proteomics technologies have numerous limitations, such as effects of epitope-changing variants, nonspecific binding, and uncertainty about target specificity. However, the many biologically relevant associations that have been derived using data from the Olink and other affinity proteomics platforms suggest that these concerns are of minor relevance. The fact that 39 novel *cis*-pQTLs were identified in this study provides further confirmatory evidence for the target specificity of their respective affinity binders.

STAR★METHODS

Detailed methods are provided in the online version of this paper and include the following:

- KEY RESOURCES TABLE
- RESOURCE AVAILABILITY
  - Lead contact
  - Materials availability
  - Data and code availability
- EXPERIMENTAL MODEL AND SUBJECT DETAILS
- METHOD DETAILS
- QUANTIFICATION AND STATISTICAL ANALYSIS
- ADDITIONAL RESOURCES

SUPPLEMENTAL INFORMATION

Supplemental information can be found online at https://doi.org/10.1016/j.xgen.2024.100506.

ACKNOWLEDGMENTS

I thank all UKB participants for their contribution. K.S. is supported by the Biomedical Research Program at Weill Cornell Medicine in Qatar, a program funded by the Qatar Foundation. K.S. is also supported by Qatar National Research Fund (QNRF) grant NPRP11C-0115-180010. Graphical abstract created with BioRender.com. The statements made herein are solely the responsibility of the author.

AUTHOR CONTRIBUTIONS

K.S. conceived the study, conducted the data analyses, and wrote the paper.

DECLARATION OF INTERESTS

The author declares no competing interests.

Received: August 4, 2023
Revised: October 25, 2023
Accepted: January 26, 2024
Published: February 24, 2024

REFERENCES

1. Suhre, K., McCarthy, M.I., and Schwenk, J.M. (2021). Genetics meets proteomics: perspectives for large population-based studies. Nat. Rev. Genet. *22*, 19–37. https://doi.org/10.1038/s41576-020-0268-2.
2. Sun, B.B., Maranville, J.C., Peters, J.E., Stacey, D., Staley, J.R., Blackshaw, J., Burgess, S., Jiang, T., Paige, E., Surendran, P., et al. (2018). Genomic atlas of the human plasma proteome. Nature *558*, 73–79. https://doi.org/10.1038/s41586-018-0175-2.
3. Folkersen, L., Gustafsson, S., Wang, Q., Hansen, D.H., Hedman, Å.K., Schork, A., Page, K., Zhernakova, D.V., Wu, Y., Peters, J., et al. (2020). Genomic and drug target evaluation of 90 cardiovascular proteins in 30,931 individuals. Nat. Metab. *2*, 1135–1148. https://doi.org/10.1038/s42255-020-00287-2.
4. Emilsson, V., Ilkov, M., Lamb, J.R., Finkel, N., Gudmundsson, E.F., Pitts, R., Hoover, H., Gudmundsdottir, V., Horman, S.R., Aspelund, T., et al. (2018). Co-regulatory networks of human serum proteins link genetics to disease. Science (New York, N.Y.) *361*, 769–773. https://doi.org/10.1126/science.aaq1327.
5. Suhre, K., Arnold, M., Bhagwat, A.M., Cotton, R.J., Engelke, R., Raffler, J., Sarwath, H., Thareja, G., Wahl, A., DeLisle, R.K., et al. (2017). Connecting genetic risk to disease end points through the human blood plasma proteome. Nat. Commun. *8*, 14357. https://doi.org/10.1038/ncomms14357.
6. Sun, B.B., Chiou, J., Traylor, M., Benner, C., Hsu, Y.-H., Richardson, T.G., Surendran, P., Mahajan, A., Robins, C., Vasquez-Grinnell, S.G., et al. (2022). Genetic regulation of the human plasma proteome in 54,306 UK Biobank participants. Preprint at bioRxiv. https://doi.org/10.1101/2022.06.17.496443.
7. Suhre, K., and Gieger, C. (2012). Genetic variation in metabolic phenotypes: study designs and applications. Nat. Rev. Genet. *13*, 759–769. https://doi.org/10.1038/nrg3314.
8. Kastenmüller, G., Raffler, J., Gieger, C., and Suhre, K. (2015). Genetics of human metabolism: an update. Hum. Mol. Genet. *24*, R93–R101. https://doi.org/10.1093/hmg/ddv263.
9. Krumsiek, J., Suhre, K., Evans, A.M., Mitchell, M.W., Mohney, R.P., Milburn, M.V., Wägele, B., Römisch-Margl, W., Illig, T., Adamski, J., et al. (2012). Mining the unknown: a systems approach to metabolite identification combining genetic and metabolic information. PLoS Genet. *8*, e1003005. https://doi.org/10.1371/journal.pgen.1003005.
10. Krumsiek, J., Suhre, K., Illig, T., Adamski, J., and Theis, F.J. (2011). Gaussian graphical modeling reconstructs pathway reactions from high-throughput metabolomics data. BMC Syst. Biol. *5*, 21. https://doi.org/10.1186/1752-0509-5-21.
11. Petersen, A.K., Krumsiek, J., Wägele, B., Theis, F.J., Wichmann, H.E., Gieger, C., and Suhre, K. (2012). On the hypothesis-free testing of metabolite ratios in genome-wide and metabolome-wide association studies. BMC Bioinf. *13*, 120. https://doi.org/10.1186/1471-2105-13-120.
12. Suhre, K., Shin, S.Y., Petersen, A.K., Mohney, R.P., Meredith, D., Wägele, B., Altmaier, E., CARDIoGRAM; Deloukas, P., Erdmann, J., et al. (2011). Human metabolic individuality in biomedical and pharmaceutical research. Nature *477*, 54–60. https://doi.org/10.1038/nature10354.
13. Shin, S.Y., Fauman, E.B., Petersen, A.K., Krumsiek, J., Santos, R., Huang, J., Arnold, M., Erte, I., Forgetta, V., Yang, T.P., et al. (2014). An atlas of genetic influences on human blood metabolites. Nat. Genet. *46*, 543–550. https://doi.org/10.1038/ng.2982.
14. Surendran, P., Stewart, I.D., Au Yeung, V.P.W., Pietzner, M., Raffler, J., Wörheide, M.A., Li, C., Smith, R.F., Wittemans, L.B.L., Bomba, L., et al. (2022). Rare and common genetic determinants of metabolic individuality and their effects on human health. Nat. Med. *28*, 2321–2332. https://doi.org/10.1038/s41591-022-02046-0.
15. Krumsiek, J., Stückler, F., Suhre, K., Gieger, C., Spector, T.D., Soranzo, N., Kastenmüller, G., and Theis, F.J. (2016). Network-based metabolite ratios for an improved functional characterization of genome-wide association study results. Preprint at bioRxiv. https://doi.org/10.1101/048512.
16. Sheils, T.K., Mathias, S.L., Kelleher, K.J., Siramshetty, V.B., Nguyen, D.-T., Bologa, C.G., Jensen, L.J., Vidović, D., Koleti, A., Schürer, S.C., et al. (2021). TCRD and Pharos 2021: mining the human proteome for disease biology. Nucleic Acids Res. *49*, D1334–D1346. https://doi.org/10.1093/nar/gkaa993.
17. Staley, J.R., Blackshaw, J., Kamat, M.A., Ellis, S., Surendran, P., Sun, B.B., Paul, D.S., Freitag, D., Burgess, S., Danesh, J., et al. (2016). PhenoScanner: a database of human genotype-phenotype associations. Bioinformatics *32*, 3207–3209. https://doi.org/10.1093/bioinformatics/btw373.
18. Hollingworth, P., Sweet, R., Sims, R., Harold, D., Russo, G., Abraham, R., Stretton, A., Jones, N., Gerrish, A., Chapman, J., et al. (2012). Genome-wide association study of Alzheimer's disease with psychotic symptoms. Mol. Psychiatr. *17*, 1316–1327. https://doi.org/10.1038/mp.2011.125.
19. Lu, Z., and Hunter, T. (2009). Degradation of activated protein kinases by ubiquitination. Annu. Rev. Biochem. *78*, 435–475. https://doi.org/10.1146/annurev.biochem.013008.092711.
20. Liu, J.Z., van Sommeren, S., Huang, H., Ng, S.C., Alberts, R., Takahashi, A., Ripke, S., Lee, J.C., Jostins, L., Shah, T., et al. (2015). Association analyses identify 38 susceptibility loci for inflammatory bowel disease and highlight shared genetic risk across populations. Nat. Genet. *47*, 979–986. https://doi.org/10.1038/ng.3359.
21. Häuser, F., Rossmann, H., Laubert-Reh, D., Wild, P.S., Zeller, T., Müller, C., Neuwirth, S., Blankenberg, S., and Lackner, K.J. (2015). Inflammatory bowel disease (IBD) locus 12: is glutathione peroxidase-1 (GPX1) the relevant gene? Gene Immun. *16*, 571–575. https://doi.org/10.1038/gene.2015.35.
22. Boughton, A.P., Welch, R.P., Flickinger, M., VandeHaar, P., Taliun, D., Abecasis, G.R., and Boehnke, M. (2021). LocusZoom.js: interactive and embeddable visualization of genetic association study results. Bioinformatics *37*, 3017–3018. https://doi.org/10.1093/bioinformatics/btab186.
23. Wallace, C. (2021). A more accurate method for colocalisation analysis allowing for multiple causal variants. PLoS Genet. *17*, e1009440. https://doi.org/10.1371/journal.pgen.1009440.
24. Giambartolomei, C., Vukcevic, D., Schadt, E.E., Franke, L., Hingorani, A.D., Wallace, C., and Plagnol, V. (2014). Bayesian test for colocalisation between pairs of genetic association studies using summary statistics. PLoS Genet. *10*, e1004383. https://doi.org/10.1371/journal.pgen.1004383.
25. Illig, T., Gieger, C., Zhai, G., Römisch-Margl, W., Wang-Sattler, R., Prehn, C., Altmaier, E., Kastenmüller, G., Kato, B.S., Mewes, H.W., et al. (2010). A genome-wide perspective of genetic variation in human metabolism. Nat. Genet. *42*, 137–141. https://doi.org/10.1038/ng.507.
26. Lagace, T.A., Curtis, D.E., Garuti, R., McNutt, M.C., Park, S.W., Prather, H.B., Anderson, N.N., Ho, Y.K., Hammer, R.E., and Horton, J.D. (2006). Secreted PCSK9 decreases the number of LDL receptors in hepatocytes and in livers of parabiotic mice. J. Clin. Invest. *116*, 2995–3005. https://doi.org/10.1172/jci29383.
27. Lagace, T.A. (2014). PCSK9 and LDLR degradation: regulatory mechanisms in circulation and in cells. Curr. Opin. Lipidol. *25*, 387–393. https://doi.org/10.1097/mol.0000000000000114.
28. Zhang, J., Dutta, D., Köttgen, A., Tin, A., Schlosser, P., Grams, M.E., Harvey, B., CKDGen Consortium; Yu, B., Boerwinkle, E., et al. (2022). Plasma proteome analyses in individuals of European and African ancestry identify cis-pQTLs and models for proteome-wide association studies. Nat. Genet. *54*, 593–602. https://doi.org/10.1038/s41588-022-01051-w.
29. Olbei, M., Thomas, J.P., Hautefort, I., Treveil, A., Bohar, B., Madgwick, M., Gul, L., Csabai, L., Modos, D., and Korcsmaros, T. (2021). CytokineLink: A Cytokine Communication Map to Analyse Immune Responses-Case Studies in Inflammatory Bowel Disease and COVID-19. Cells *10*. https://doi.org/10.3390/cells10092242.
30. Macian, F. (2005). NFAT proteins: key regulators of T-cell development and function. Nat. Rev. Immunol. *5*, 472–484. https://doi.org/10.1038/nri1632.

CellPress
OPEN ACCESS

31. Lee, J.U., Kim, L.K., and Choi, J.M. (2018). Revisiting the Concept of Targeting NFAT to Control T Cell Immunity and Autoimmune Diseases. Front. Immunol. *9*, 2747. https://doi.org/10.3389/fimmu.2018.02747.

32. Gao, R., Zhang, Y., Zeng, C., and Li, Y. (2022). The role of NFAT in the pathogenesis and targeted therapy of hematological malignancies. Eur. J. Pharmacol. *921*, 174889. https://doi.org/10.1016/j.ejphar.2022.174889.

33. Metzelder, S.K., Michel, C., von Bonin, M., Rehberger, M., Hessmann, E., Inselmann, S., Solovey, M., Wang, Y., Sohlbach, K., Brendel, C., et al. (2015). NFATc1 as a therapeutic target in FLT3-ITD-positive AML. Leukemia *29*, 1470–1477. https://doi.org/10.1038/leu.2015.95.

34. Wang, Q., Zhou, Y., Jackson, L.N., Johnson, S.M., Chow, C.W., and Evers, B.M. (2011). Nuclear factor of activated T cells (NFAT) signaling regulates PTEN expression and intestinal cell differentiation. Mol. Biol. Cell *22*, 412–420. https://doi.org/10.1091/mbc.E10-07-0598.

35. Szklarczyk, D., Gable, A.L., Lyon, D., Junge, A., Wyder, S., Huerta-Cepas, J., Simonovic, M., Doncheva, N.T., Morris, J.H., Bork, P., et al. (2019). STRING v11: protein-protein association networks with increased coverage, supporting functional discovery in genome-wide experimental datasets. Nucleic Acids Res. *47*, D607-d613. https://doi.org/10.1093/nar/gky1131.

36. Bennett, J., and Starczynowski, D.T. (2022). IRAK1 and IRAK4 as emerging therapeutic targets in hematologic malignancies. Curr. Opin. Hematol. *29*, 8–19. https://doi.org/10.1097/moh.0000000000000693.

37. Zhande, R., Dauphinee, S.M., Thomas, J.A., Yamamoto, M., Akira, S., and Karsan, A. (2007). FADD negatively regulates lipopolysaccharide signaling by impairing interleukin-1 receptor-associated kinase 1-MyD88 interaction. Mol. Cell Biol. *27*, 7394–7404. https://doi.org/10.1128/mcb.00600-07.

38. King, C.L., Adams, J.H., Xianli, J., Grimberg, B.T., McHenry, A.M., Greenberg, L.J., Siddiqui, A., Howes, R.E., da Silva-Nunes, M., Ferreira, M.U., and Zimmerman, P.A. (2011). Fy(a)/Fy(b) antigen polymorphism in human erythrocyte Duffy antigen affects susceptibility to Plasmodium vivax malaria. Proc. Natl. Acad. Sci. USA *108*, 20113–20118. https://doi.org/10.1073/pnas.1109621108.

39. Moskovitz, R., Pholcharee, T., DonVito, S.M., Guloglu, B., Lowe, E., Mohring, F., Moon, R.W., and Higgins, M.K. (2023). Structural basis for DARC binding in reticulocyte invasion by Plasmodium vivax. Nat. Commun. *14*, 3637. https://doi.org/10.1038/s41467-023-39357-w.

40. Nibbs, R.J.B., and Graham, G.J. (2013). Immune regulation by atypical chemokine receptors. Nat. Rev. Immunol. *13*, 815–829. https://doi.org/10.1038/nri3544.

41. von Hundelshausen, P., Agten, S.M., Eckardt, V., Blanchet, X., Schmitt, M.M., Ippel, H., Neideck, C., Bidzhekov, K., Leberzammer, J., Wichapong, K., et al. (2017). Chemokine interactome mapping enables tailored intervention in acute and chronic inflammation. Sci. Transl. Med. *9*, eaah6650. https://doi.org/10.1126/scitranslmed.aah6650.

42. Hopkins, A.L. (2008). Network pharmacology: the next paradigm in drug discovery. Nat. Chem. Biol. *4*, 682–690. https://doi.org/10.1038/nchembio.118.

43. Altan-Bonnet, G., and Mukherjee, R. (2019). Cytokine-mediated communication: a quantitative appraisal of immune complexity. Nat. Rev. Immunol. *19*, 205–217. https://doi.org/10.1038/s41577-019-0131-x.

44. Dhillon, B.K., Smith, M., Baghela, A., Lee, A.H.Y., and Hancock, R.E.W. (2020). Systems Biology Approaches to Understanding the Human Immune System. Front. Immunol. *11*, 1683. https://doi.org/10.3389/fimmu.2020.01683.

45. Schäfer, J., and Strimmer, K. (2005). A shrinkage approach to large-scale covariance matrix estimation and implications for functional genomics. Statistical Applications in Genetics and Molecular Biology, *4*, Article32. https://doi.org/10.2202/1544-6115.1175.

46. Stelzer, G., Rosen, N., Plaschkes, I., Zimmerman, S., Twik, M., Fishilevich, S., Stein, T.I., Nudel, R., Lieder, I., Mazor, Y., et al. (2016). The GeneCards Suite: From Gene Data Mining to Disease Genome Sequence Analyses. Curr. Protoc. Bioinformatics *54*, 1, 30.31-31.30.33. https://doi.org/10.1002/cpbi.5.

47. Purcell, S., Neale, B., Todd-Brown, K., Thomas, L., Ferreira, M.A.R., Bender, D., Maller, J., Sklar, P., de Bakker, P.I.W., Daly, M.J., and Sham, P.C. (2007). PLINK: a tool set for whole-genome association and population-based linkage analyses. Am. J. Hum. Genet. *81*, 559–575. https://doi.org/10.1086/519795.

48. Bycroft, C., Freeman, C., Petkova, D., Band, G., Elliott, L.T., Sharp, K., Motyer, A., Vukcevic, D., Delaneau, O., O'Connell, J., et al. (2018). The UK Biobank resource with deep phenotyping and genomic data. Nature *562*, 203–209. https://doi.org/10.1038/s41586-018-0579-z.

49. Band, G., and Marchini, J. (2018). BGEN: a binary file format for imputed genotype and haplotype data. Preprint at bioRxiv. https://doi.org/10.1101/308296.

STAR★METHODS

KEY RESOURCES TABLE

REAGENT or RESOURCE	SOURCE	IDENTIFIER
Deposited data		
pQTL summary statistics from the UKB PPP project (Table S6 of Sun et al.[6])	Sun et al.[6]	https://doi.org/10.1101/2022.06.17.496443
Genomics, proteomics, and phenotype data of UK Biobank participants	UK Biobank	https://biobank.ndph.ox.ac.uk/showcase/
Summary statistics using imputed UKB genotype data for the regions (+/−500kb) around the 8,462 rQTLs discovered in the GWAS	This study	https://doi.org/10.6084/m9.figshare.23695398
Manhattan plots for the GWAS (PDF format)	This study	https://doi.org/10.6084/m9.figshare.23695398
Regional association plots for the local refinements (PDF format)	This study	https://doi.org/10.6084/m9.figshare.23695398
Software and algorithms		
R	The R Project for Statistical Computing	https://www.r-project.org
Rstudio	Posit	https://posit.co
Genomics analysis tools	DNAnexus	https://ukbiobank.dnanexus.com/landing
GeneNet	Schäfer and Strimmer[45]	N/A
Other		
STRING	Szklarczyk et al.[35]	https://string-db.org
NCBI Pharos	Sheils et al.[16]	https://pharos.nih.gov
PhenoScanner	Staley et al.[17]	http://www.phenoscanner.medschl.cam.ac.uk
CytokineLink	Olbei et al.[29]	https://github.com/korcsmarosgroup/CytokineLink
LocusZoom	Boughton et al.[22]	http://locuszoom.sph.umich.edu
GeneCards	Stelzer et al.[46]	https://www.genecards.org
Plink	Purcell et al.[47]	https://www.cog-genomics.org/plink/2.0

RESOURCE AVAILABILITY

Lead contact

Further information and requests for resources and reagents should be directed to and will be fulfilled by the lead contact, Karsten Suhre (kas2049@qatar-med.cornell.edu).

Materials availability

This study did not generate new unique reagents.

Data and code availability

- All analyzed data was from UK Biobank and obtained through the UKB RAP system under application reference number 43418 and is accessible upon application online via https://biobank.ndph.ox.ac.uk/showcase/.
- Full summary statistics for all 2,821 GWAS with ratios using the array-genotyped UKB data shall be made available via the GWAS catalog.
- Summary statistics using imputed UKB genotype data for the regions (+/−500kb) around the 8,462 rQTLs discovered in the GWAS are available on FigShare (https://doi.org/10.6084/m9.figshare.23695398), both for the discovery and the replication cohort.
- Manhattan and regional association plots based on this data are available in PDF format on FigShare at the same URL.
- This study did not generate any unique code.

CellPress
OPEN ACCESS

EXPERIMENTAL MODEL AND SUBJECT DETAILS

All data and samples were collected by UK Biobank following all relevant ethical guidelines and procedures and were shared with the UKB users under rules reviewed by the UK Biobank ethics committee and board.

All data was obtained through the UKB RAP system on the DNAnexus platform (data dispensed on April 23, 2023; application id 43418) for samples satisfying the criterion "Number of proteins measured | Instance 0" is greater than "0" (https://biobank.ndph.ox.ac.uk/showcase/field.cgi?id=30900). Imputed genotypes[48] were extracted from BGEN files (https://biobank.ndph.ox.ac.uk/showcase/label.cgi?id=100319) using bgenix[49] and reformatted to text format (.raw) using plink[47] for further analysis in R. Phenotype data for age, sex, BMI, the first three genotype principal components, and the classification of genetic ethnic grouping were extracted using the DNAnexus cohort browser and the table downloader app (https://ukbiobank.dnanexus.com/landing). Details on genomics and proteomics data QC and preprocessing are available in the accompanying UK Biobank resource files (available at the respective showcase links given above).

The downloaded proteomics dataset comprised NPX values for 1,463 proteins for 52,749 participants. NPX values correspond to relative protein concentrations and are reported on a log-scale. Data analysis was restricted to 52,705 samples that were collected at baseline (instance 0). Samples were split into a discovery set of 43,509 samples identified as Caucasian based on the genetic ethnic grouping variable and a replication set of 9,196 ethnically diverse samples (https://biobank.ndph.ox.ac.uk/showcase/field.cgi?id=22006). A total of 5,717 unique variants corresponding to 10,248 pQTLs of the UKB PPP GWAS were analyzed. These pQTLs were obtained from Table S6 of Sun et al.[6]

METHOD DETAILS

Partial correlations were computed using the R function ggm.estimate.pcor from the package GeneNet.[45] All baseline samples were used for this step. As this analysis does not allow for the presence of missing values, samples with more than 20% missing protein values were removed (N = 1,840), followed by proteins that were missing in more than 20% of the samples (N = 3). The remaining missing data points were imputed to minimum (N = 37,419). A total of 11,936 partial correlations were identified at a Bonferroni significance cut-off p value of 4.7×10^{-8}. The smallest r^2 at this level was 0.00176.

The GWAS on 2,821 ratios was conducted using plink2[47] on the UKB RAP platform hosted by DNAnexus with the –glm option, using age, sex, and the first the genoPCs as covariates. We used the array-genotyped UKB data with the following variant filtering options: –geno 0.1, –hwe 1e−15, –mac 100, –maf 0.01, –mind 0.1.

QUANTIFICATION AND STATISTICAL ANALYSIS

Linear models with inverse-normal scaled proteomics data (NPX values) as dependent variables and genotype, age, sex, and the first three genotype principal components were computed using the R function "lm". For ratios, inverse-normal scaled differences between the two NPX values were used, based on the relation log(A/B) = log(A) - log(B) and the NPX values representing protein levels on a log-scale. The p-gain for associations with ratios between two protein traits was computed as the smaller of the two p values for the individual trait associations divided by the p value for the ratio association.[11] Log10-scaled p values and p-gains were used throughout to avoid numeric overflows and rounding of small p values to zero.

For all pairs of proteins with a Bonferroni significant partial correlation all variants that were associated with at least one of the two proteins in a pQTL in the UKB PPP GWAS were identified. For these variant – protein pairs the single protein and ratio association statistics were computed using the discovery and replication samples separately.

ADDITIONAL RESOURCES

The STRING database of proteins and their functional interactions was used to identify known relationships between proteins.[35] The database was downloaded from https://string-db.org/cgi/download (version 11.5, accessed 5 May 2023). Annotated cytokine and cytokine-receptor pairs were downloaded from CytokineLink[29] (https://github.com/korcsmarosgroup/CytokineLink, accessed 30 June 2023). Drug target development status was obtained from NCBI Pharos[16] (accessed 11 July 2023). Variants were annotated with PhenoScanner API[17] using proxies based on EUR LD $r^2 > 0.8$ (accessed 5 May - 11 July 2023). LocusZoom[22] was used to generate regional association plots with LD annotation (EUR population). GeneCards[46] was used to obtain general information about the associated genes and proteins.

Cell Genomics, Volume *4*

Supplemental information

Genetic associations with ratios between protein levels detect new pQTLs and reveal protein-protein interactions

Karsten Suhre

SUPPLEMENTAL MATERIAL

Genetic associations with ratios between protein levels detect new pQTLs and reveal protein-protein interactions.

Karsten Suhre[1,2,*]

[1] Bioinformatics Core, Weill Cornell Medicine-Qatar, Education City, 24144 Doha, Qatar

[2] Department of Biophysics and Physiology, Weill Cornell Medicine, New York, NY, U.S.A.

[*] Correspondence to K.S. (kas2049@qatar-med.cornell.edu)

SUPPLEMENTAL FIGURES

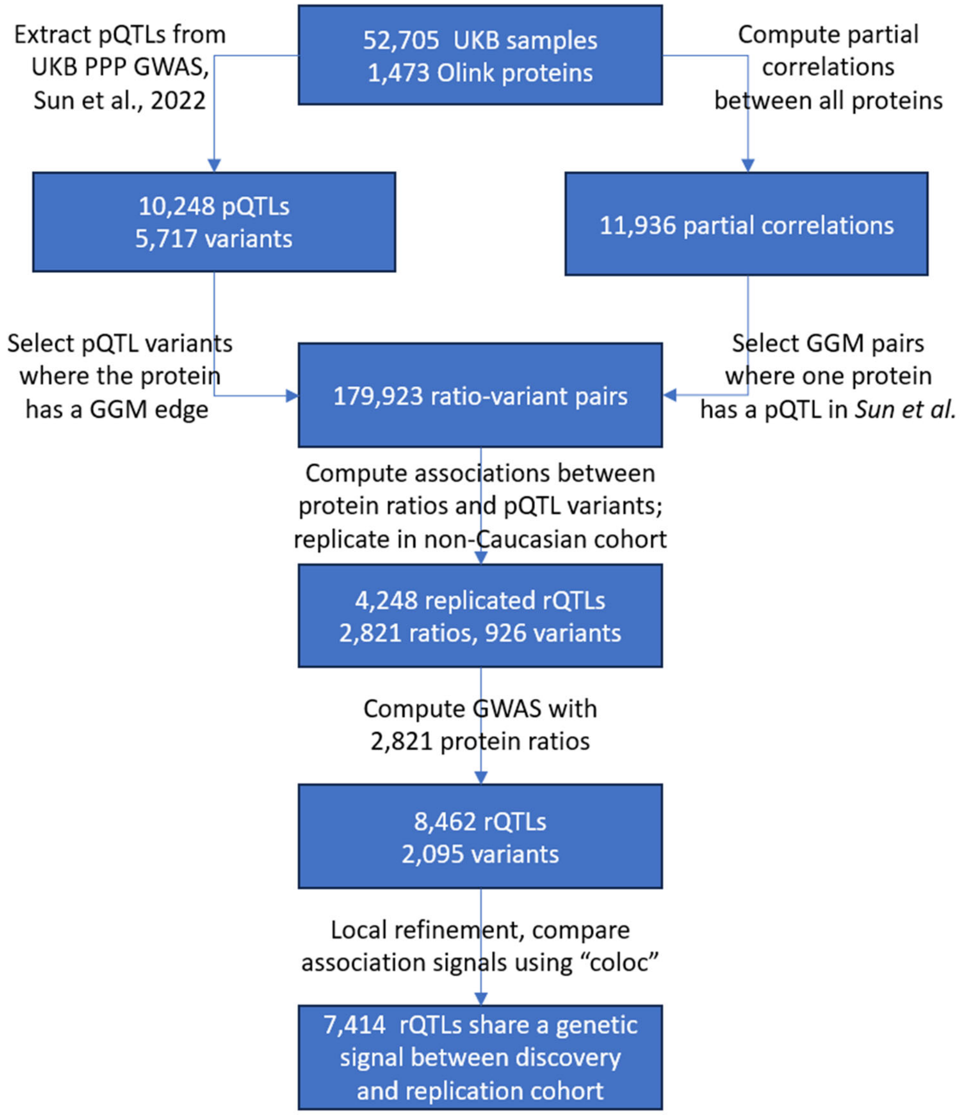

Figure S1: Flowchart of the study, related to Figures 1-2

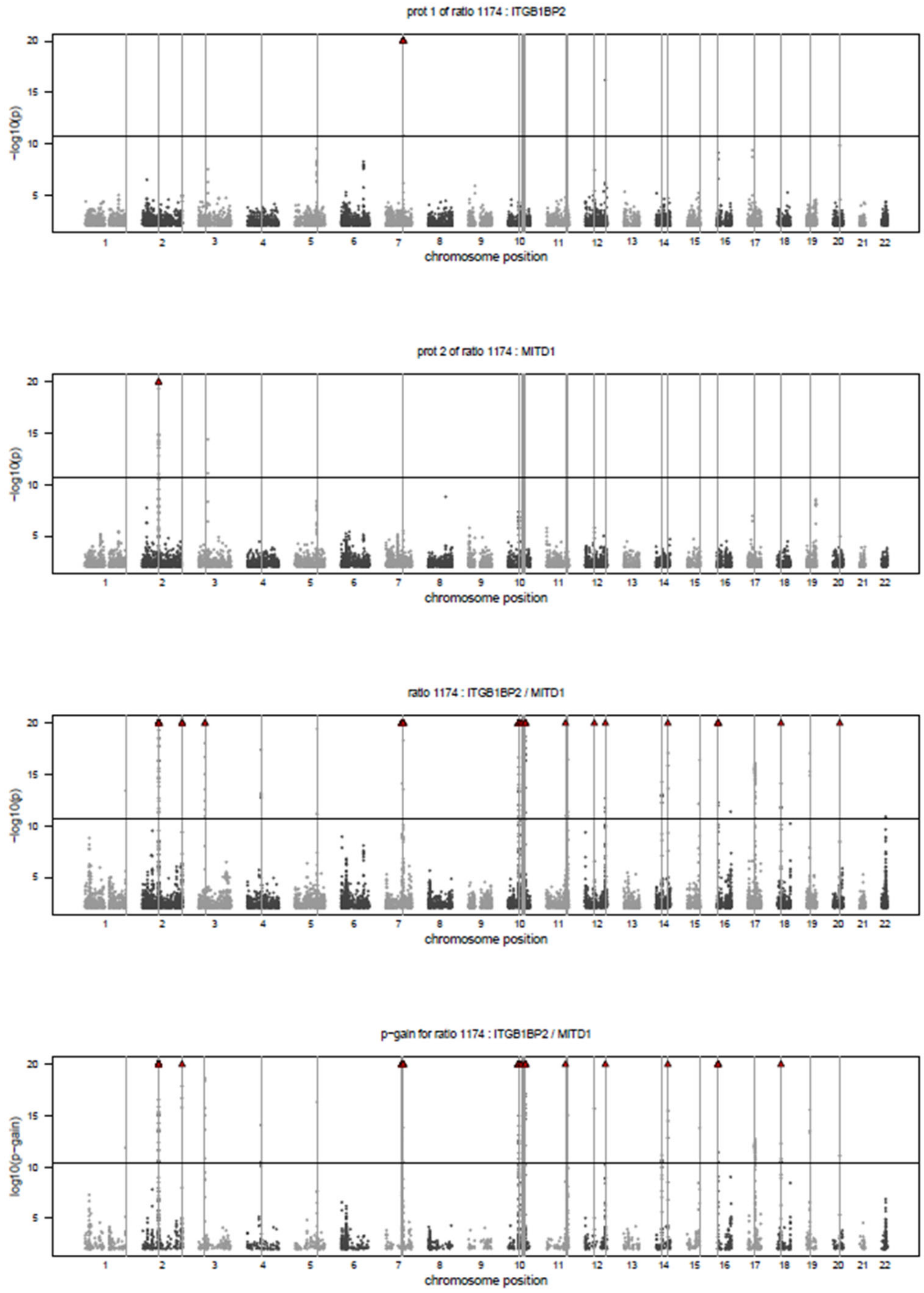

Figure S2: Example of Manhattan plots for a ratio, related to Figures 1-2. This Figure explains how by using ratios novel genetic loci can be uncovered. Plotted are the associations of the two individual proteins (ITGB1BP2 and MTD1), the ratio (ITGB1BP2 / MTD1), and the p-gain of the ratio using array genotype data; Similar Manhattan plots are available in PDF format for the 2,821 ratios on FigShare (doi:10.6084/m9.figshare.23695398).

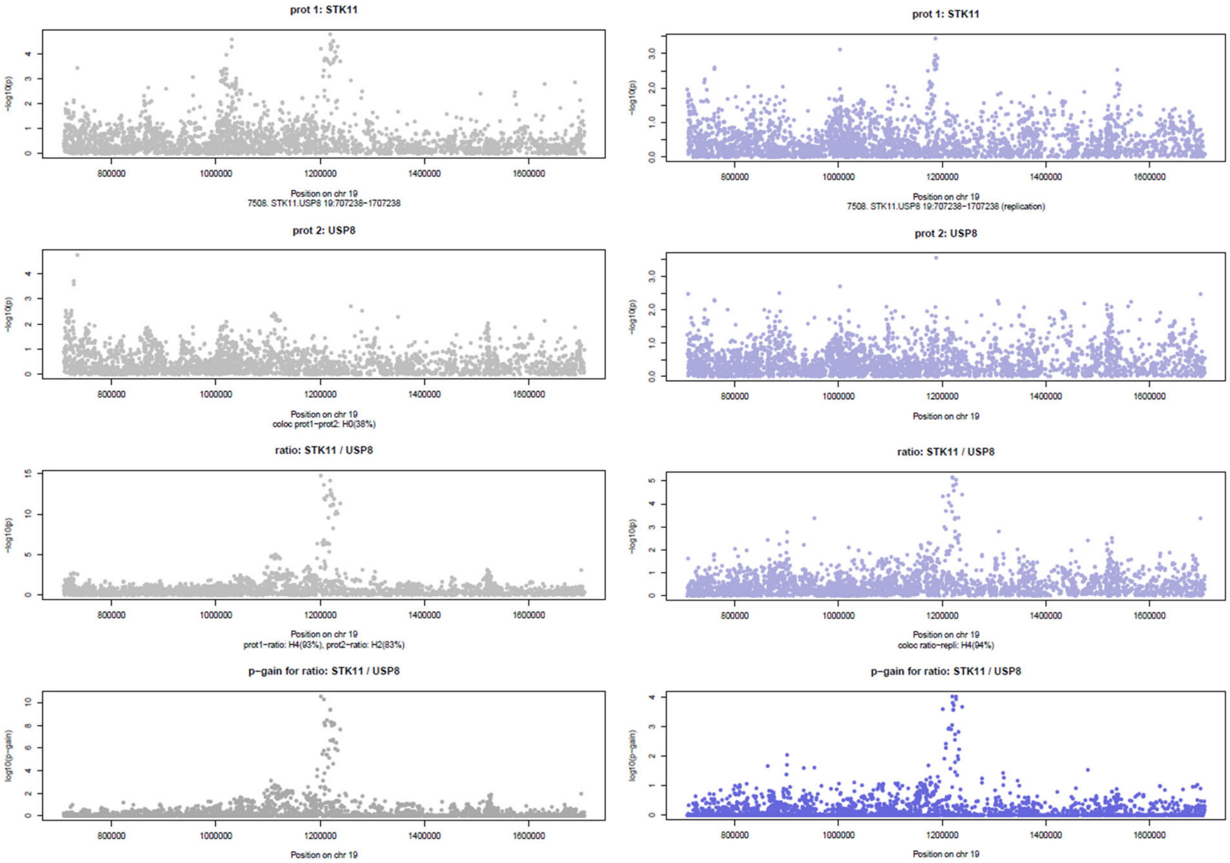

(a) (b)

Figure S3: Example of regional association plots for rQTLs, related to Figures 1-2. This Figure explains how by using ratios a genetic signal can emerge from the noise. Plotted are the associations of the two individual proteins (STK11 and USP8), the ratio (STK11 / USP8), and the p-gain of the ratio using imputed genotype data +/-500kb around the variant rs3764640 for the discovery (a) and the replication cohort (b); The subtitles indicate the most likely *coloc* hypotheses regarding the similarity between the relevant genetic signals; Similar regional association plots together with the full summary statistics used in these plots for 8,462 rQTLs are available in PDF format on FigShare (doi:10.6084/m9.figshare.23695398).

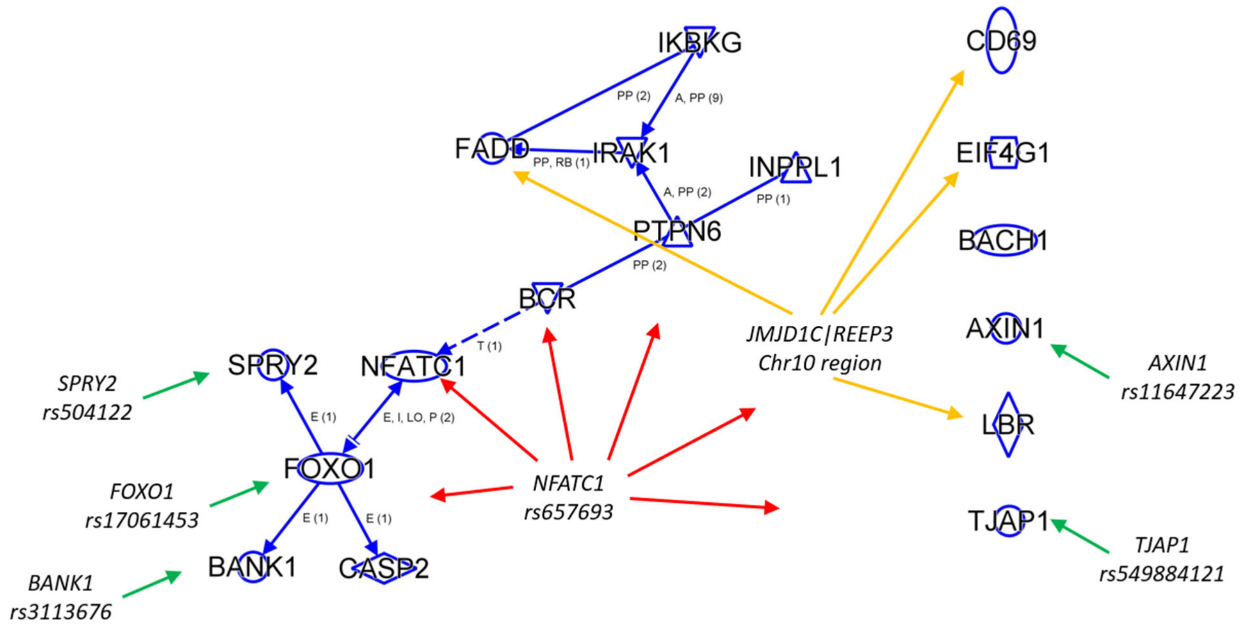

Figure S4: NFATC1 network, related to Figures 1-2. Protein-protein interactions obtained using Ingenuity Pathway Analysis (IPA)'s connect function with default settings (accessed 4 July 2023); Blue arrows indicate IPA interactions with the following abbreviations: A: activation, E: expression, I: inhibition, LO: localization, P: phosphorylation, PP: protein-protein interaction, RB: regulation of binding, T: transcription; Numbers in parentheses indicate multiple sources of evidence; Green arrows: rQTLs for the ratio of NFATC1 with the respective proteins at a *cis*-location; Orange: multiple variants on Chr10 around 65 MB associated with ratios between the listed proteins and NFATC1; Red: rs657693 at the *NFATC1* gene locus associated with all depicted proteins in a ratio with NFATC1 (details are in Table S4).

	CCL13	CCL8	CCL7	CCL2	CCL26	CXCL8	CCL11	CCL3	CCL14	CXCL6	TGFB1	PDGFA	HGF	THPO	TNFSF13	CXCL12	CXCL1	CCL17	CCL4	CXCL11	TNFSF14	IL7
CCL13	-230.0	542.9	500.1	0.0	0.0	0.0	0.0	0.0	0.0	0.0	75.7	66.0	0.0	57.8	28.8	20.5	0.0	0.0	0.0	4.2	10.7	0.0
CCL8	-542.9	179.8	0.0	-335.7	-291.1	-226.0	-106.0	-54.3	-98.1	-87.7	-54.1	0.0	-4.6	-36.0	-16.9	0.0	-3.1	-5.7	-14.5	0.0	0.0	0.0
CCL7	-500.1	0.0	337.3	-410.0	-274.5	-147.4	-122.7	-116.5	-108.2	0.0	-29.6	0.0	-57.9	0.0	-13.9	0.0	0.0	0.0	0.0	0.0	0.0	0.0
CCL2	0.0	335.7	410.0	-291.1	0.0	0.0	0.0	0.0	0.0	0.0	0.0	0.0	0.0	0.0	2.3	0.0	0.0	0.0	0.0	0.0	0.0	0.0
CCL26	0.0	291.1	274.5	0.0	-182.8	0.0	0.0	0.0	0.0	0.0	28.5	5.8	14.9	17.5	19.3	12.5	0.0	0.0	0.0	0.0	0.0	0.0
CXCL8	0.0	226.0	147.4	0.0	0.0	-89.4	0.0	0.0	0.0	0.0	8.0	0.0	0.0	1.5	8.4	0.0	0.0	0.0	0.0	0.0	0.0	0.0
CCL11	0.0	106.0	122.7	0.0	0.0	0.0	-33.8	0.0	0.0	0.0	0.0	0.0	0.0	0.0	0.0	0.0	0.0	0.0	0.0	0.0	0.0	0.0
CCL3	0.0	54.3	116.5	0.0	0.0	0.0	0.0	-14.5	0.0	0.0	0.0	0.0	0.0	0.0	1.4	0.0	0.0	0.0	0.0	0.0	0.0	0.0
CCL14	0.0	98.1	108.2	0.0	0.0	0.0	0.0	0.0	-31.5	0.0	0.0	0.0	0.0	0.0	5.2	0.0	0.0	0.0	0.0	0.0	0.0	0.0
CXCL6	0.0	87.7	0.0	0.0	0.0	0.0	0.0	0.0	0.0	-32.2	12.1	7.5	0.0	5.1	3.5	0.9	0.0	0.0	0.0	11.7	3.1	10.3
TGFB1	-75.7	54.1	29.6	0.0	-28.5	-8.0	0.0	0.0	0.0	-12.1	-0.2	0.0	0.0	0.0	0.1	0.0	-6.1	-5.4	-0.1	0.9	0.0	0.1
PDGFA	-66.0	0.0	0.0	0.0	-5.8	0.0	0.0	0.0	0.0	-7.5	0.0	-0.3	0.0	0.0	0.1	0.0	-3.3	-9.8	0.0	0.0	0.0	0.2
HGF	0.0	4.6	57.9	0.0	-14.9	0.0	0.0	0.0	0.0	0.0	0.0	0.0	-1.1	0.0	0.2	0.0	0.0	0.0	0.0	1.0	0.0	0.0
THPO	-57.8	36.0	0.0	0.0	-17.5	-1.5	0.0	0.0	0.0	-5.1	0.0	0.0	0.0	-0.4	0.1	0.0	-2.6	-5.4	0.0	0.7	0.0	0.0
TNFSF13	-28.8	16.9	13.9	-2.3	-19.3	-8.4	0.0	-1.4	-5.2	-3.5	-0.1	-0.1	-0.2	-0.1	0.2	-0.1	-2.1	-2.6	-0.9	0.0	-0.1	0.0
CXCL12	-20.5	0.0	0.0	0.0	-12.5	0.0	0.0	0.0	0.0	-0.9	0.0	0.0	0.0	0.0	0.1	-0.1	0.0	-1.1	0.0	0.1	0.0	0.0
CXCL1	0.0	3.1	0.0	0.0	0.0	0.0	0.0	0.0	0.0	0.0	6.1	3.3	0.0	2.6	2.1	0.0	-20.4	0.0	0.0	5.8	2.3	8.5
CCL17	0.0	5.7	0.0	0.0	0.0	0.0	0.0	0.0	0.0	0.0	5.4	9.8	0.0	5.4	2.6	1.1	0.0	-17.9	0.0	8.4	2.9	5.1
CCL4	0.0	14.5	0.0	0.0	0.0	0.0	0.0	0.0	0.0	0.0	0.1	0.0	0.0	0.0	0.9	0.0	0.0	0.0	-3.5	2.3	0.0	0.0
CXCL11	-4.2	0.0	0.0	0.0	0.0	0.0	0.0	0.0	0.0	-11.7	-0.9	0.0	-1.0	-0.7	0.0	-0.1	-5.8	-8.4	-2.3	2.0	-0.3	0.0
TNFSF14	-10.7	0.0	0.0	0.0	0.0	0.0	0.0	0.0	0.0	-3.1	0.0	0.0	0.0	0.0	0.1	0.0	-2.3	-2.9	0.0	0.3	-0.3	0.1
IL7	0.0	0.0	0.0	0.0	0.0	0.0	0.0	0.0	0.0	-10.3	-0.1	-0.2	0.0	0.0	0.0	0.0	-8.5	-5.1	0.0	0.0	-0.1	0.1

Figure S5: P-gain matrix for the association of rs12075 with all ratios between cytokines, related to Figures 1-2. SNP rs12075 (1:159175354:G:A) is an amino acid changing variant in ACKR1 aka DARC (c.125G>A, p.Gly42Asp) and defines the co-dominant Duffy blood type alleles Fy^a (Gly) and Fy^b (Asp). Limited to associations with -log10(p-value) > 10 or log10(p-gain) > 10 (full matrix in Supplementary Table 6); Values on the diagonal are -log10(p-value) for the single protein associations; Values in the off-diagonal cells are -log10(p-gain); The directionality of the associations with the copy number of the Fy^a allele are indicated by the sign and colored red (negative association) and green (positive association). Note that the -log10(p-value) for the ratios can be obtained by adding the log10(p-gain) of the ratio to the larger of the two -log10(p-value) of the single protein associations (full data in Table S9).
